# A molecular safeguard in *Escherichia coli* Braun’s lipoprotein (Lpp) biogenesis

**DOI:** 10.1128/mbio.01845-26

**Published:** 2026-09-17

**Authors:** Emily J. LaMarre, Makayla R. Braunlin, Brian Kloss, Faiza Rafi, Bogdan I. Iorga, Anuradha Janakiraman

**Affiliations:** 1 Department of Biology, The City College of CUNY 14770, New York, New York, USA; 2 Program in Biology, The Graduate Center, CUNY, New York, New York, USA; 3 New York Structural Biology Center 535965, New York, New York, USA; 4 Université Paris-Saclay, CNRS UPR 2301, Institut de Chimie des Substances Naturelles (ICSN) 57474, Gif-sur-Yvette, France; University of Georgia, Athens, Georgia, USA

**Keywords:** cell envelope, lipoproteins, *Escherichia coli*, Lpp, DcrB

## Abstract

Braun’s lipoprotein (Lpp) is the most abundant protein present in *Escherichia coli*. Lpp is an outer membrane (OM) lipoprotein that contributes to the mechanical integrity of the cell envelope by tethering the OM to the underlying peptidoglycan (PGN). It has long been known that Lpp accumulation at the inner membrane (IM) is toxic due to aberrant tethering of the IM to the PGN. However, it is not known if there exists a cellular mechanism that mitigates such anomalous Lpp-mediated linkages. Here, we report the identification of DcrB, an inner membrane lipoprotein, as a molecular safeguard of Lpp biogenesis in *E. coli*. We demonstrate that DcrB promotes *E. coli* viability under conditions of lipoprotein maturation stress, and that in its absence Lpp-PGN linkages are altered. Genetic and biochemical tests reveal that DcrB’s function is in promoting the efficiency of Lpp maturation, likely through a direct interaction. Overall, our results are consistent with two, albeit not mutually exclusive, roles for DcrB in Lpp biogenesis: promoting Lpp maturation and ameliorating the formation of deleterious IM-Lpp-PGN linkages.

**IMPORTANCE:**

Lpp, an outer membrane (OM) lipoprotein, was discovered more than five decades ago as the first member of the bacterial lipoprotein family, which constitutes a group of important cell envelope proteins. Lpp crosslinks the OM to the underlying peptidoglycan (PGN) and contributes to the structural integrity of the cell envelope. However, Lpp-mediated crosslinks from the inner membrane (IM) to the PGN are toxic. Here, we identify an IM lipoprotein, DcrB, that binds Lpp and improves the efficiency of Lpp’s journey across the IM. In so doing, DcrB attenuates the formation of aberrant IM-PGN linkages. Overall, this study reveals the existence of a critical safeguard in the biogenesis of Lpp, a paradigmatic bacterial lipoprotein.

## INTRODUCTION

The distinguishing feature of gram-negative bacteria is the multi-layered cell envelope consisting of two membranes, inner and outer (IM and OM), delimiting an aqueous periplasmic compartment containing a thin peptidoglycan (PGN) layer (1). This distinctive envelope architecture contributes to the shape and mechanical properties of the cell, provides selective permeability to nutrients, and offers protection against toxic agents (1). Lipoproteins are an important and diverse class of envelope proteins that participate in a wide range of functions, including the maintenance of the double-membraned cell envelope architecture (2–6). They are anchored to either membrane by characteristic N-terminally linked fatty acyl chains (2, 3).

**Fig 1:**
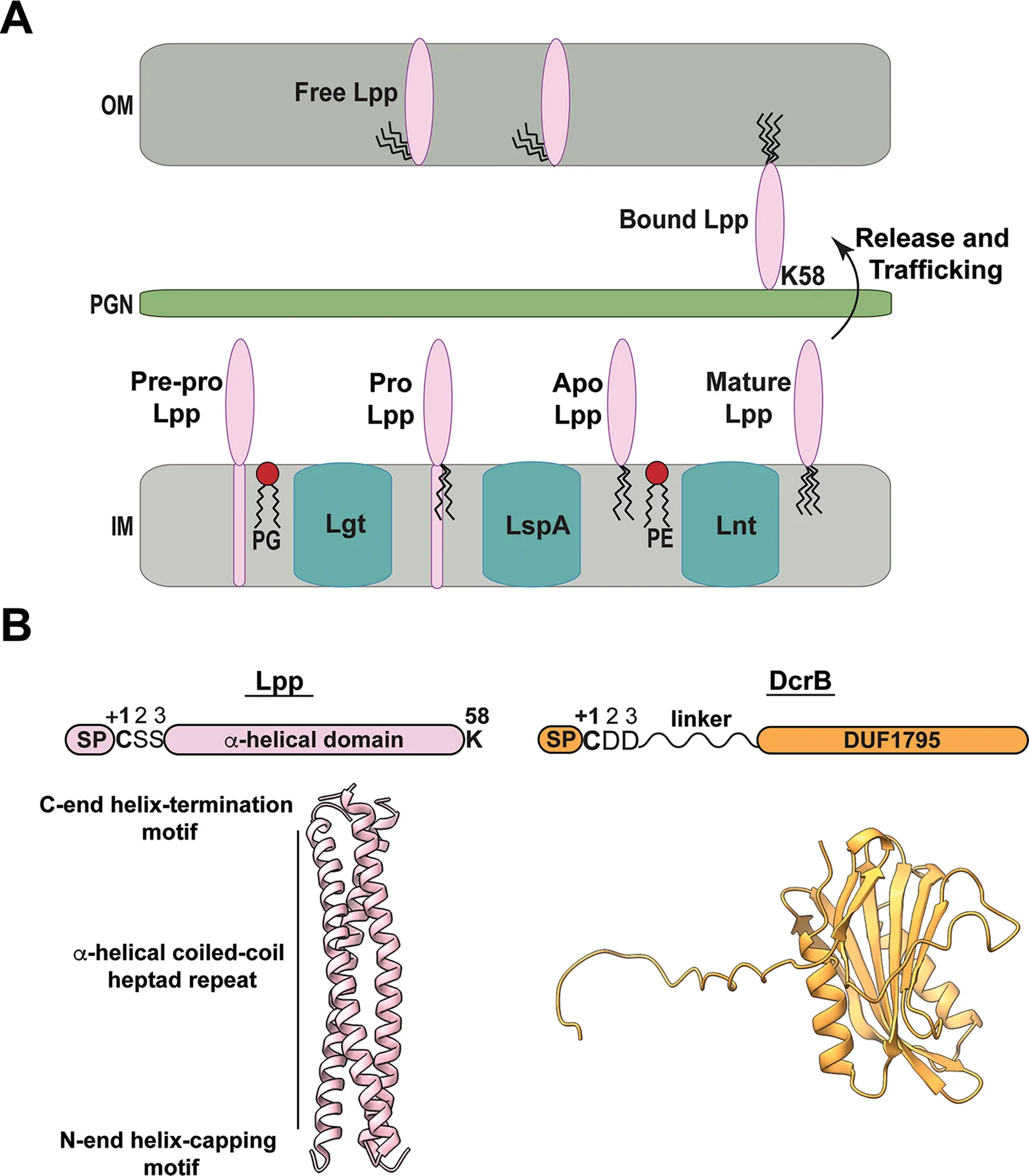
Lpp maturation and structures of Lpp and DcrB. (**A**) Lpp biogenesis in *E. coli* is shown. Secreted pre-pro-Lpp is acylated and modified at the IM by three essential enzymes, Lgt, LspA, and Lnt. Triacylated mature Lpp is extracted from the IM and trafficked to the OM by the Lol pathway (details not shown). Lpp resides in the OM either as bound-Lpp, which is tethered to the underlying PGN via the C-terminal lysine (K58) residue, or as free-Lpp that traverses the OM and is surface-exposed. IM, inner membrane; OM, outer membrane; PE, phosphatidylethanolamine; PG, phosphatidylglycerol; PGN, peptidoglycan. (**B**) The domain architecture of Lpp is illustrated: SP, signal sequence; the conserved cysteine (+1); serine residues at +2, +3 sorting positions; the helical domain with the C-terminal lysine residue (K58). The crystal structure of the mature Lpp trimer (PDB: 1EQ7) lacking the C1 and K58 residues is shown with the helix termination motifs flanking the α-helical coiled-coil repeat. The domain organization of DcrB is illustrated: SP, signal sequence; the conserved cysteine (+1); Asp-dependent IM retention at +2, +3 sorting positions; disordered linker; and the DUF1795 domain. An AlphaFold-predicted structure of the *E. coli* protein (AF-P0AEE1-F1-v4) without the signal peptide residues (1–19) is shown.

Lipoproteins are synthesized in the cytoplasm with a signature N-terminal signal sequence containing a four-residue lipobox terminating in an invariant cysteine residue (2, 7). This cysteine becomes the first amino acid residue (Cys^+1^) of the mature protein and is acylated during lipoprotein modification (2, 8). Upon traversing the IM via the Sec or Tat translocation machinery, precursor lipoproteins are post-translationally modified at the IM with the covalent attachment of acyl chains and cleavage of the signal peptide (2). In *E. coli*, this modification, commonly referred to as the lipoprotein maturation pathway, occurs through the sequential actions of three essential integral IM enzymes (9, 10) (Fig. 1A). Lgt, a diacylglyceryl transferase, adds a diacylglyceryl moiety to the Cys^+1^ residue of the prepro-lipoprotein to form a pro-lipoprotein. LspA, a conserved type II signal peptidase, then cleaves the signal peptide to generate an apo-lipoprotein, a rate-limiting step in the pathway (11). Finally, Lnt, an N-acyltransferase, adds the third acyl chain to the newly freed amino terminus of Cys^+1^, leading to the formation of the mature triacylated protein (8, 11–13). At this stage, distinguished by the sorting signals (amino acid residues at the +2 and +3 positions relative to Cys^+1^ and the length of a disordered linker), lipoproteins either remain in the IM or are targeted to the OM via the Lol trafficking machinery (2, 3, 6, 14–16). Lipoproteins that lack the “Lol avoidance” sorting signal (most commonly lacking an aspartate at the +2 position) are extracted from the IM by the ABC transporter LolCDE, transferred to LolA, which ferries them across the periplasm and delivers them to LolB, an OM lipoprotein that facilitates their assembly into the OM (4, 6, 14, 15, 17–20). In certain mutant backgrounds, LolA and LolB are dispensable, suggesting the presence of an alternate trafficking route (21). Most lipoproteins are localized to the OM where they are present facing the periplasm or are surface exposed (2, 3, 15).

The paradigmatic member of bacterial lipoproteins is the OM Braun’s lipoprotein, or Lpp, which was discovered more than 56 years ago (22–24). Lpp plays a key role in maintaining the envelope architecture by tethering the OM to the underlying PGN (24) (Fig. 1A). The Lpp-facilitated OM-PGN linkages contribute to maintaining the periplasmic width, periplasmic pressure, and stiffness of the cell envelope (25–27). Proper periplasmic width controls the overall architecture and integrity of the cell envelope by contributing to envelope size, signal transduction across the periplasm, and assembly of trans-envelope protein machinery (25, 28). At ~1 million copies per cell, Lpp is the most abundant protein in *E. coli* (29). It is a small (~6 kDa) protein that adopts a trimeric conformation in the OM, and via its carboxy-terminal lysine (K58) provides the only covalent link from the OM to the PGN (17, 30–36) (Fig. 1B). In exponentially growing *E. coli*, approximately one-third of cellular Lpp, referred to as the “bound form,” is present in the periplasm and covalently attached to the PGN (37). Nearly all of the remaining Lpp spans the OM and is surface exposed (the “free form”) (36) (Fig. 1A). A crystal structure of Lpp (residues 2–57 of the mature protein, lacking the acyl chains, the signal peptide, and Cys^+1^) shows a trimeric α-helical coiled-coil with two helix-capping motifs (30, 33, 34) (Fig. 1B). However, it is unclear whether this structure is representative of the physiological conformation of both forms of Lpp, bound and free, which are considered to be in dynamic equilibrium in the cell (36, 38). Although deletion of *lpp* does not affect *E. coli* viability under standard laboratory growth conditions, mutant cells reveal OM permeability defects, increased OM vesiculation, leakage of periplasmic contents, cell separation, and attenuated virulence, confirming the importance of Lpp in cell envelope integrity (24, 39, 40).

When lipoprotein biogenesis is inhibited, Lpp accumulates in the IM and establishes toxic IM-PGN links (41). Untethering Lpp from the PGN by deleting the K58 residue of Lpp suppresses this toxicity, indicating that the primary cause of cell death is the linkage of the IM to the PGN (41, 42). However, the question as to what prevents an abundant cargo such as Lpp from aberrantly linking to the PGN during lipoprotein modification at the IM remains unresolved. Under standard growth conditions, the efficiency of lipoprotein modification may be robust enough to obviate the need for regulation. We wondered whether this was also the case under conditions of inefficient lipoprotein biogenesis, or whether, under those conditions, a molecular safeguard would be needed to prevent IM Lpp from establishing toxic links to the PGN.

Here, we describe the characterization of an essential safety mechanism of Lpp biogenesis under conditions of lipoprotein maturation stress. This regulation is mediated by DcrB, an IM lipoprotein of previously unknown function that bears the structurally similar DUF1795 functional domain, known to be present in chaperones or chaperone-like proteins that bind hydrophobic ligands in other bacteria (43–45) (Fig. 1B and Fig. S1). Our results, described below, are consistent with a model in which DcrB binds Lpp and promotes its efficient processing at the IM during growth in maturation stress conditions. A corollary of this function lies in ameliorating the formation of deleterious links of Lpp from the IM to the PGN.

## RESULTS

### Suppressor analysis of *dcrB* null toxicity implicates a role for DcrB in Lpp biogenesis

**Fig 2:**
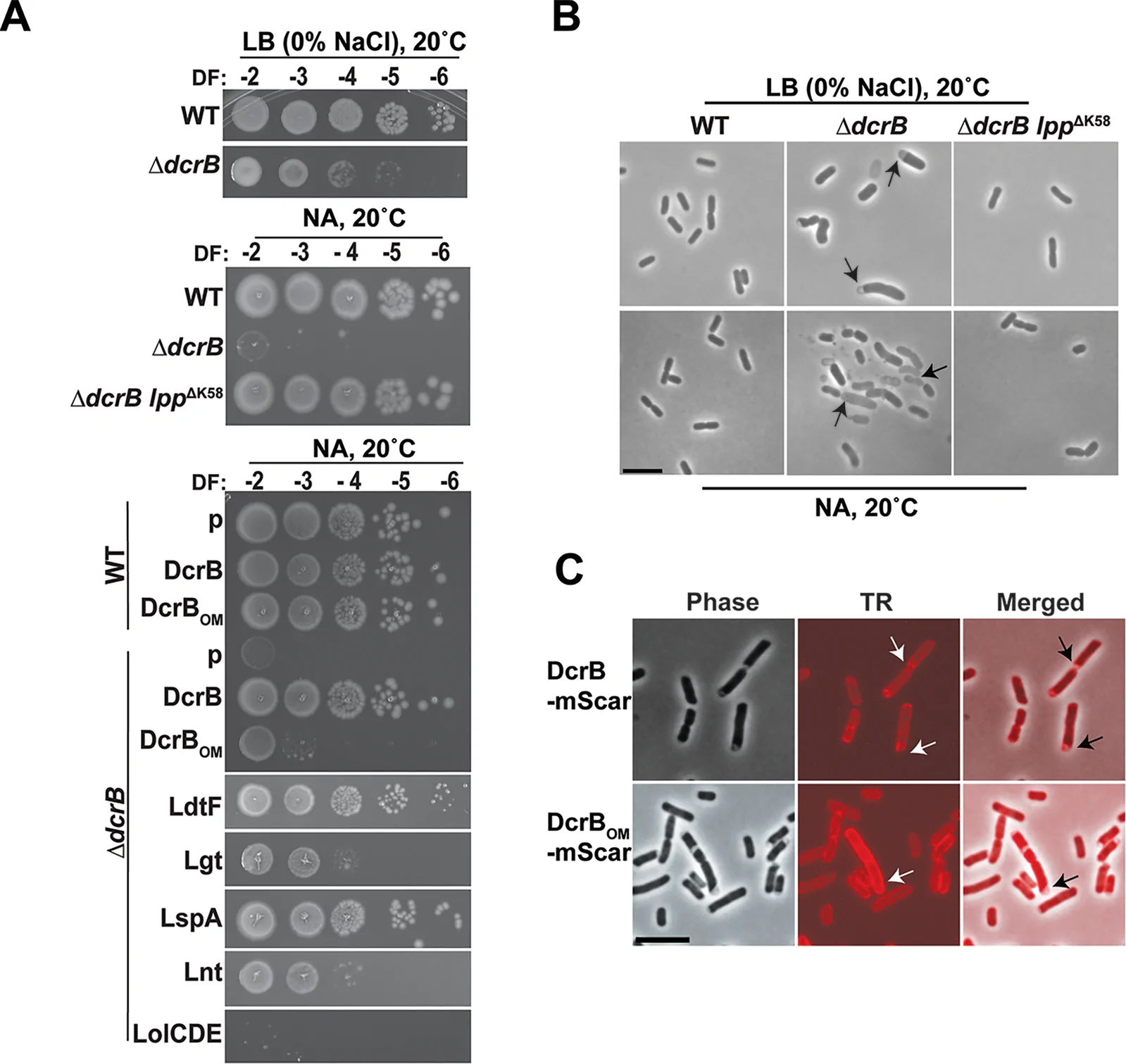
Genetic interactions, cell morphology, and localization of *dcrB*. (**A**) Overnight cultures of WT and isogenic mutant strains *dcrB* (AM135) and *dcrB lpp*^ΔK58^ (AM1714) were normalized to OD_600_, and aliquots of serial dilutions were spotted on LB (0% NaCl) or NA plates and incubated for 72 h at 20°C prior to imaging. For plasmid-bearing strains (in sequential order, pDSW206 vector [(p] for WT or Δ*dcrB* [AM135] or pDSW206 expressing DcrB [pEL3}, DcrB_OM_ [pEL4], Lgt [pAM8], LspA [pEL8], Lnt [pEL9], and LolCDE [pEL10], and pTrc99a expressing LdtF [pFR1]), the agar medium was supplemented with Amp and IPTG at a final concentration of 100 μM for pDSW206, or 1,000 μM for pTrc99a, and grown on NA plates for 60 h at 20°C. Representative plate images are shown. (**B**) Overnight cultures of WT and isogenic mutant derivatives were grown in permissive conditions of LB (1% NaCl) at 37°C and washed in LB (0% NaCl) or NB before being subcultured in the same medium. Cells were grown at 20°C in LB (0% NaCl) or NB before being visualized on 2% agarose pads by phase microscopy. Arrows point to regions of cytoplasmic shrinkage and IM retraction or lysis. Bar = 5 µm. (**C**) Phase, fluorescence (TxRED), and merged images of DcrB and DcrB_OM_ variants fused to mScarlet-I (pEL5 and pEL6) in the TB28 background are shown. The mScar fusion variants were induced with IPTG at 100 μM final concentration. Arrows reveal the fluorescence of the fusion variants in the membranes around plasmolysis bays. Bar = 5 µm.

We first identified *dcrB* (*yhhR*) in our laboratory as one half of a synthetic lethal gene pair (*yciB dcrB; yciB* encodes an IM protein implicated in membrane homeostasis) and subsequently showed that *dcrB* is essential for *E. coli* growth in low salt (LB [0% NaCl] or nutrient agar [NA]) and low temperature (20°C) conditions (43, 46). Note that *dcrB* null grows worse in NA relative to LB (0% NaCl), presumably due to the lower osmolyte content in NA (Fig. 2A). *dcrB* mutant cell death could be complemented by expressing *dcrB in trans* (43) (Fig. 2A). Mutant toxicity could also be suppressed by deletion of *lpp* or *ldtB* (an L,D-transpeptidase known to be the major linker of Lpp and PGN) or by introduction of *lpp*^ΔK58^ at the chromosomal locus of *lpp*, consistent with Lpp forms accumulating in the IM and establishing lethal links to the PGN (43) (Fig. 2A). Here, we confirmed these results and extended them to show that unlinking of Lpp by overexpression of LdtF/DpaA, an enzyme that breaks Lpp’s covalent linkage to the PGN, also suppressed *dcrB* null cell death (47, 48) (Fig. 2A). This corroborated an earlier report where increased expression of LdtF partially restored the viability of a *yciB dcrB* double mutant where Lpp establishes lethal IM-PGN crosslinks (47). The morphological changes exhibited by *dcrB* null cells under restrictive conditions included cytoplasmic shrinkage, IM retraction, and cell lysis, reminiscent of cells with aberrant Lpp-PGN links from the IM (43) (Fig. 2B). The genetic and microscopy results implied that PGN-linked Lpp forms accumulate in the IM of *dcrB* null cells under restrictive growth conditions.

Next, we tested whether the IM localization of DcrB was important for its function. We created a *dcrB* variant (*dcrB_OM_*) whose sorting sequence is altered such that it is re-routed to the OM and confirmed the OM localization by employing a cell biological assay in which cells expressing the DcrB_OM_ variant fused to mScarlet-I (mScar) were subjected to plasmolysis. This leads to the contraction of the cytoplasm and the concomitant retraction of the IM, creating plasmolysis bays (49). The fluorescence patterns of DcrB-mScar and DcrB_OM_-mScar fusions within plasmolysis bays confirmed that each variant localized to its expected destination, the IM and OM, respectively (Fig. 2C). While expression of the *dcrB_OM_* derivative did not affect WT growth, it failed to suppress *dcrB* toxicity, suggesting the physiological relevance of the IM-location of DcrB (Fig. 2A).

### DcrB promotes growth under impaired lipoprotein maturation conditions

Earlier, we had shown that overexpression of Lgt, the essential diacylglyceryl transferase mediating the first step of lipoprotein maturation, promoted partial growth of the *dcrB* mutant under restrictive conditions, implying that lipoprotein maturation may be inefficient in this environment (43). To test whether subsequent steps of lipoprotein maturation and/or trafficking play a role in the IM accumulation of Lpp in *dcrB* null cells, LspA, Lnt, and LolCDE were overexpressed *in trans* and tested for growth in the non-permissive condition. Increased levels of LspA restored robust growth to the *dcrB* null while that of Lnt supported modest restoration of growth, and overexpression of LolCDE failed to improve growth (43) (Fig. 2A). LspA levels remained essentially the same in *dcrB*^+/−^ cells under restrictive growth, revealing that mutant toxicity was not due to altered LspA amounts (Fig. S2). These results suggest that the accumulation of PGN-linked IM Lpp in the *dcrB* null under non-permissive growth conditions results from defects in lipoprotein maturation rather than in extraction from the IM.

To clarify this inference, we tested the viability of *dcrB* null cells in conditions of impaired lipoprotein maturation via established chemical and genetic methods. First, we examined whether depletion of Lgt led to reduced growth of *dcrB* null cells relative to *dcrB^+^* in standard growth conditions. As expected, in the presence of glucose, the *lgt* depletion strain was not viable (10). In *lgt*-replete conditions, with inducer supplementation that was sufficient to sustain growth of the conditional *lgt* mutant, *dcrB* null grew comparatively worse (Fig. S3A).

Next, we examined whether *dcrB* null cells displayed altered sensitivity in the presence of globomycin (Glb), an inhibitor of LspA activity (50–52). MIC assays revealed that relative to WT, *dcrB* null cells were twofold more sensitive to Glb (Table 1). This was not due to altered OM barrier properties of the *dcrB* null as revealed by the limited entry of SDS-EDTA, a known marker of OM permeability defects (Fig. S4). As expected, cells lacking *lpp* were more resistant to the drug since the lethal accumulation of Lpp-mediated IM-PGN links was not present (53). The *dcrB lpp* null resembled the *lpp* null with respect to resistance to Glb, suggesting that *lpp* is epistatic to *dcrB* with respect to drug sensitivity (Table 1).

**TABLE 1:** Minimum inhibitory concentrations to LspA inhibitor, globomycin (Glb), in mutant strains

Genotype	Glb (µg/mL)
WT	16
*dcrB*	8
*lpp*	64
*dcrB lpp*	64

We also monitored the growth patterns of *lgt* or *lspA* depletion in conditions (LB [0% NaCl] at 20°C) that are restrictive for the *dcrB* null. As expected, *lgt* or *lspA* depletion strains are not viable in the absence of the inducer (Fig. S3B and C). Also, as expected, expression of LspA, but not Lgt, restored robust growth to the *dcrB* null (Fig. 2A and Fig. S3B and C). Low concentrations of L-arabinose (L-Ara) supplementation, sufficient to promote growth in the *lspA* depletion strain, supported only mild growth upon the removal of *dcrB* (Fig. S3C). These results recapitulated the observations from the Lgt-depletion or LspA chemical inhibition tests, conducted in otherwise standard conditions, that diminishing lipoprotein maturation worsens the viability of the *dcrB* null (Fig. 2A, Fig. S3A, and Table 1). Deletion of *lpp* restored growth in these mutant backgrounds, underscoring the notion that *lpp* lies downstream of *dcrB* in terms of viability in these conditions (Fig. S3B and C). Overall, these results support the conclusion that *dcrB* promotes *E. coli* viability under conditions of inefficient lipoprotein maturation, and that it acts upstream of Lpp accumulation in the lipoprotein maturation pathway.

### Presence of DcrB improves viability of an IM-Lpp variant

**Fig 3:**
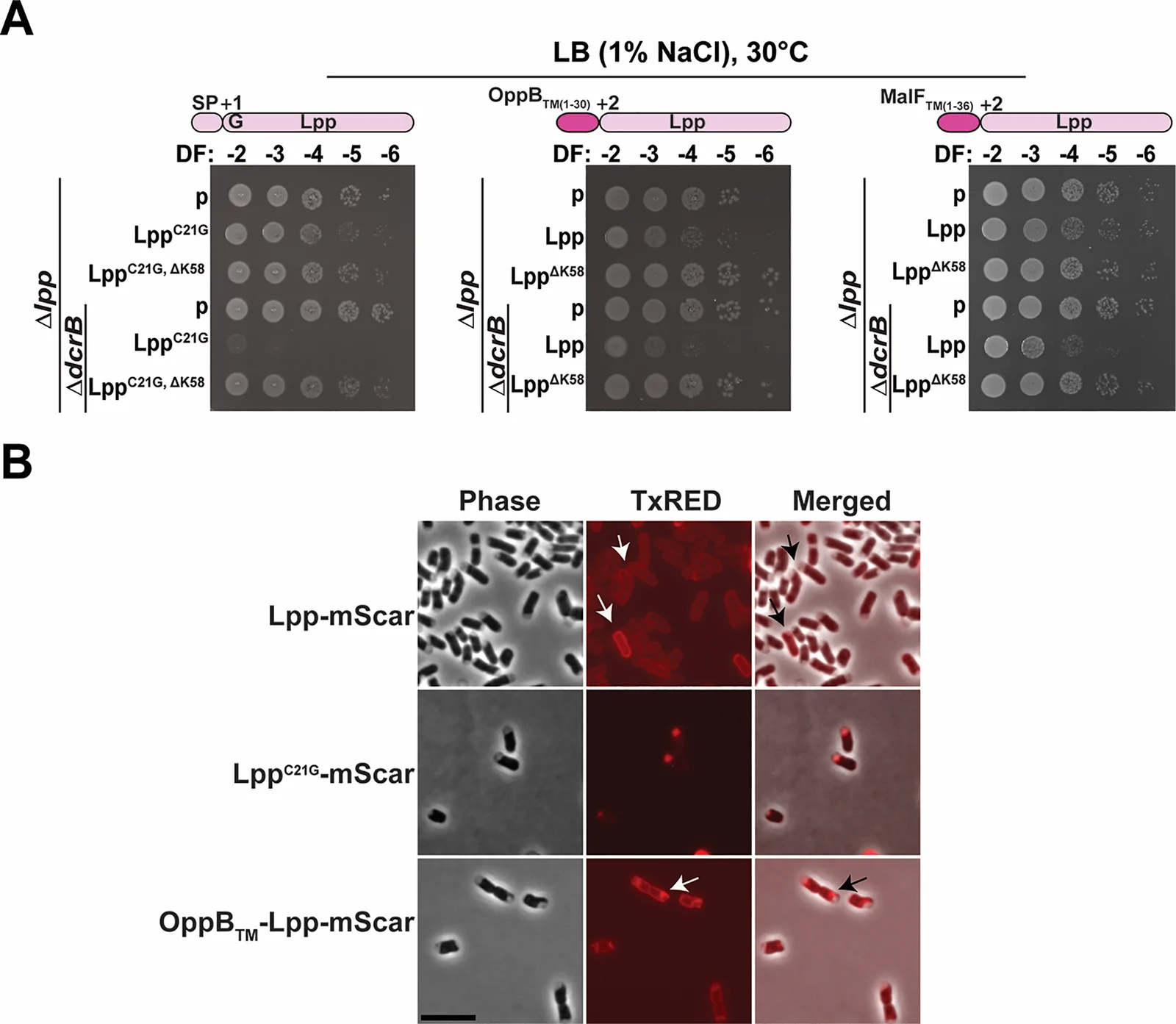
Growth and localization of IM-Lpp variants. (**A**) Viability of the Lpp^C21G^ variant, where the Cys (+1) was replaced with a glycine residue. Overnight cultures of *lpp* null expressing empty pDSW204 vector (p) in *dcrB*^+^ (AM949) or null backgrounds (AM814) or bearing pDSW204-*lpp*^C21G^ (pFR2) with or without the K58 residue (pFR3) were spotted on LB (1% NaCl) with Amp and IPTG at 250 µM final concentration and incubated for ~16–18 h at 30°C before imaging. Viability of the OppB_TM_-Lpp variant in which Lpp residues 1 through 21 were replaced with those of the OppB transmembrane domain (TM) residues 1 through 30. pDSW204-OppB_TM_-Lpp variants with (pEL13) or without the K58 residue (pEL14) were grown in *lpp* (AM949) or *dcrB lpp* (AM814) mutant backgrounds and spot-plated as described above, except IPTG inducer was added at 100 μM final concentration. Viability of the MalF_TM_-Lpp variant in which Lpp residues 1 through 21 were replaced with those of MalF’s transmembrane domain (TM) residues 1–36. Plasmids pDSW204-MalF_TM_-Lpp with (pEL15) and without (pEL16) the K58 residue were grown in *lpp* (AM949) or *dcrB lpp* (AM814) mutant backgrounds and spot-plated as described above, except IPTG inducer was added at 75 μM final concentration. A representative plate image is shown for each variant. (**B**) TB28 strains expressing either pDSW204-Lpp-mScar (pEL17), pDSW204-Lpp^C21G^-mScar (pEL42), or pDSW204-OppB_TM_-Lpp-mScar (pEL19) were subjected to plasmolysis, and localization of the fusions was visualized as described in Materials and Methods. Phase, fluorescence (TxRED), and merged images are shown. Arrows point to fluorescence localization in membranes around plasmolysis bays. Bar = 5 µm.

The results thus far were consistent with at least two possible roles for DcrB: regulating Lpp-mediated IM-PGN links and in general promoting lipoprotein transit through the maturation steps. To distinguish between the two possibilities, we deployed an Lpp variant, Lpp^C21G^, that lacked lipid tails (54) (Fig. 3A). If DcrB’s role is in mitigating IM-Lpp-PGN links, one would expect the Lpp^C21G^ variant to be less toxic in the presence of *dcrB*, even under standard conditions of growth. Indeed, this is what we observed—the growth of the Lpp^C21G^ variant was improved in *dcrB*^+^ cells (Fig. 3A). Untethering of Lpp in this background (Lpp^C21G^,^ΔK58^) restored growth, providing support that the lethality associated with Lpp^C21G^ was due to aberrant IM-PGN crosslinks (Fig. 3A). However, the predicted cleavage of Lpp^C21G^ by signal peptidase I/LepB is reported to be high (~50%, Table S1), and indeed fluorescence from an Lpp^C21G^-mScar variant was mostly localized to the plasmolysis bays (Fig. 3B). We refined our test by constructing another IM variant of Lpp, OppB_TM_-Lpp, in which Lpp’s signal sequence was replaced with the transmembrane (TM) domain of OppB, an IM protein (55) (Fig. 3A). The OppB-Lpp variant is not expected to interact with Lgt, and the predicted signal peptidase-mediated cleavage was negligible (Table S1). We confirmed the IM localization of the variant by employing the aforementioned plasmolysis bay assay (49). The fluorescence patterns of the OppB-Lpp-mScar fusion, but not of Lpp-mScar fusion, followed that of the retracted IM in the plasmolysis bays, confirming the presence of the variant at the IM (Fig. 3B). Next, the growth of OppB_TM_-Lpp was examined in cells deleted for endogenous *lpp* in *dcrB^+/−^* backgrounds. The viability of OppB_TM_-Lpp fusion was modestly enhanced by the presence of DcrB compared to cells lacking *dcrB* (Fig. 3A). The observed viability was not due to a specific property of the OppB_TM_ domain, as another transmembrane Lpp fusion, MalF_TM_-Lpp, revealed similar growth patterns in *dcrB*^+/−^ backgrounds (56) (Fig. 3A). The major cause of cell death in the IM Lpp variants was the formation of IM-PGN crosslinks, as expression of an OppB_TM_-Lpp^ΔK58^ or MalF_TM_-Lpp^ΔK58^ variant restored viability to *dcrB* null (Fig. 3A). Our results are consistent with an earlier report that Lpp lacking lipid tails can establish crosslinks to the PGN (54). Collectively, the genetic data suggested a role for DcrB in mitigating Lpp-mediated deleterious IM-PGN links.

### *dcrB* null cells reveal alterations in Lpp forms

**Fig 4:**
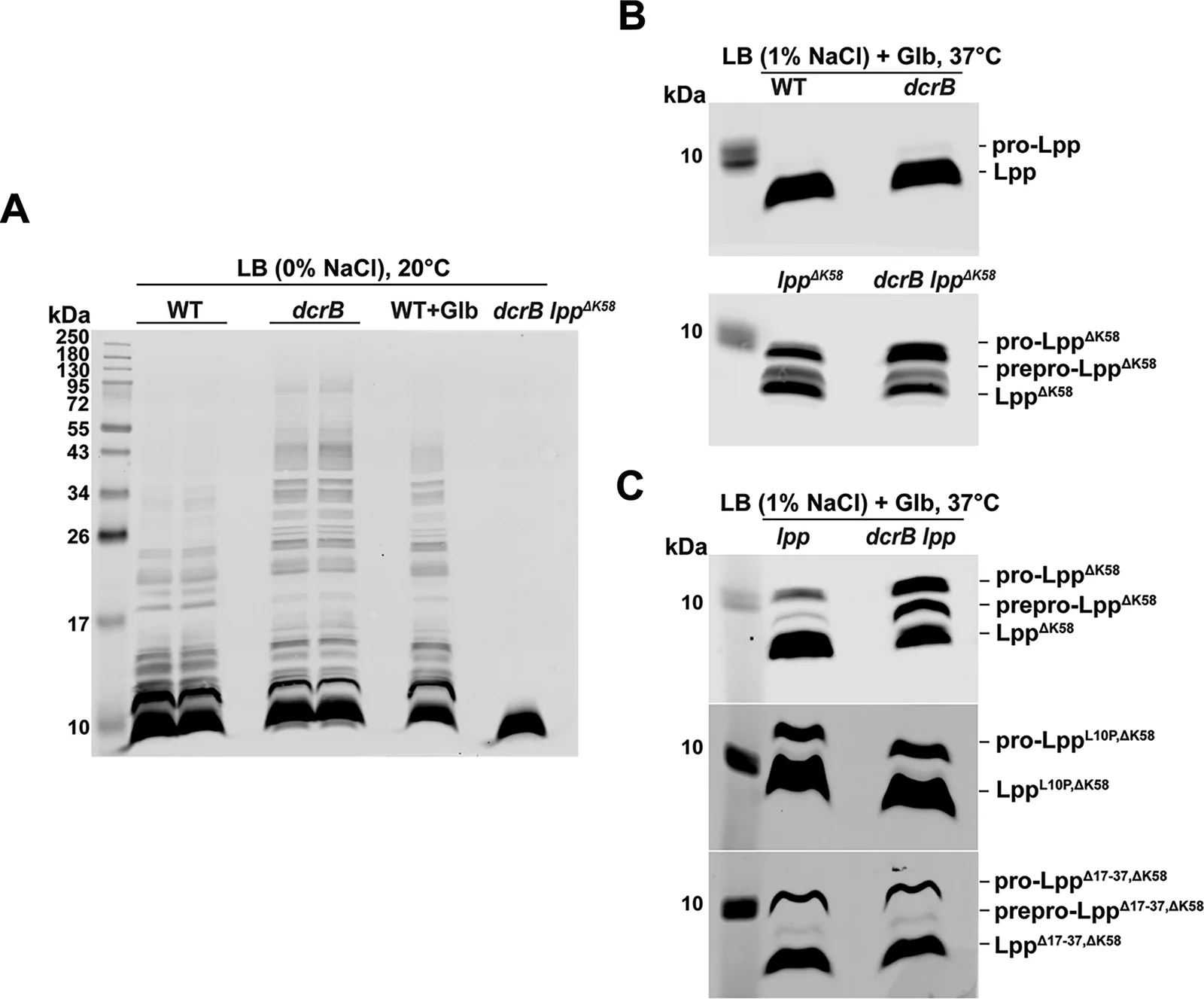
DcrB modulates Lpp-PGN links and Lpp maturation flux. (**A**) Immunoblot of Lpp forms is shown. Indicated strains (WT, AM135, and AM1714) were grown overnight in LB (1% NaCl) at 37°C, washed, and subcultured 1:100 in LB (0% NaCl) to OD_600_ of 0.03 and grown for ~18 h at 20°C. Cells were harvested and normalized to OD_600_, and proteins were extracted in the presence of lysozyme, resolved by gel electrophoresis, and immunoblotted for Lpp as described in Materials and Methods. For treatment with Glb, the inhibitor was added after ~14 h of growth at 8 μg/mL, and cells were allowed to grow for an additional ~5 h at 20°C. *dcrB* null cells grown under low salt and temperature stress relative to WT and WT cells treated with Glb are shown. Two biological replicates for WT and *dcrB* null are shown. (**B**) Overnight cultures of indicated strains were subcultured to OD_600_ ~ 0.03 in LB (1% NaCl) at 37°C with 2 μg/mL Glb (WT and AM135) or 8 μg/mL Glb (AM1712 and AM1714) and grown to mid-exponential phase. Cells were harvested, normalized to OD_600_, whole cell proteins were extracted and resolved by gel electrophoresis and immunoblotted for Lpp, as described in Materials and Methods. Half the sample volume was loaded for *lpp*^ΔK58^ strains relative to WT Lpp, as it is entirely in the free form. (**C**) Overnight cultures of *lpp* (AM949) or *dcrB lpp* (AM814) bearing plasmid-borne Lpp^ΔK58^ (pEL37), Lpp^L10P, ΔK58^ (pEL39), or Lpp^Δ17–37,ΔK58^ (pEL41) were subcultured to OD_600_ ~ 0.03 in LB (1% NaCl) with Amp and IPTG at 1 mM final concentration at 37°C with 4 μg/mL Glb and grown and processed as in panel **B**. Lpp^L10P,ΔK58^ migrated more slowly than Lpp^ΔK58^. Plasmid-borne Lpp^L10P, ΔK58^ and Lpp^Δ17–37,ΔK58^ were expressed less relative to plasmid-borne Lpp^ΔK58^ (Fig. S5). Furthermore, the Lpp^Δ17–37^ variant is not detected well by the Lpp antibody used in this study (36). For these reasons, twice the sample volumes were loaded for these strains.

The above results raised the expectation that relative to WT, *dcrB* null cells would accumulate heterogeneous Lpp forms under conditions of lipoprotein maturation stress. First, we immunoblotted for Lpp in WT and *dcrB* null cells grown in restrictive conditions (LB [0% NaCl] at 20°C). To allow for detection of PGN-bound Lpp, cells were treated with lysozyme (23, 36). WT cells revealed a ladder-like pattern of Lpp bands, characteristic of the protein bound to PGN fragments of different lengths (36, 57) (Fig. 4A). As expected, only free-form Lpp was present in cells bearing the *lpp^ΔK58^* allele (54) (Fig. 4A). Upon treatment with Glb (0.5× MIC of WT), the abundance of both low- and high-molecular-weight Lpp bands was altered in WT cells (Fig. 4A). Somewhat similar to Glb-treated cells, the *dcrB* deletion revealed greater abundance of high-molecular-weight Lpp bands (Fig. 4A). These differences were not due to changes in total protein levels (Fig. S5A).

To further investigate if DcrB indeed modulates Lpp linkages to PGN, we examined the accumulation of Lpp in *dcrB^+/−^* cells grown in standard media containing sub-inhibitory levels of Glb (0.125× MIC for WT and 0.25× for *dcrB* null, respectively). Lysozyme treatment was excluded in these preparations, ensuring the detection of primarily free-form Lpp (36). A mild increase in pro-Lpp accumulation was observed in the *dcrB* null compared to WT (Fig. 4B). These changes were not due to altered levels of total protein (Fig. S5B). Together, these findings implicated DcrB in modulating bound-form/free-form Lpp pools under conditions of inefficient lipoprotein maturation (low salt and low temperature or supplementation with Glb).

### Unhooking Lpp did not improve the maturation flux of Lpp in *dcrB* null

We reasoned that if indeed Lpp-PGN linkages contributed to the alterations in Lpp forms in the *dcrB* mutant, then levels of Lpp^ΔK58^, present entirely in the free form in cells, should remain more or less similar regardless of the presence of DcrB under conditions of Glb stress. Strikingly, we detected increased accumulation of precursor forms of Lpp^ΔK58^ in the *dcrB* null (Fig. 4B). This suggested that although unhooking Lpp from the PGN restored viability to the *dcrB* null under restrictive growth, it did not improve Lpp transit at the IM in the *dcrB* mutant. Given that the expression, lipidation, and OM localization of Lpp^ΔK58^ are similar to those of WT Lpp (41, 58), it is unlikely that these properties of Lpp^ΔΚ58^ are responsible for the observed phenotype.

Corroborative evidence was obtained from sucrose density gradient membrane fractionation results from *lpp*^ΔK58^ cells, as this strain background allows for facile separation of the two membranes (41). Cells were grown in NB at low temperature conditions, and cell lysates were subjected to sucrose density centrifugation. IM fractions were demarcated by NADH oxidase activity, which is restricted to this membrane (Fig. S6). As expected, Lpp^ΔK58^ was present mostly in the OM fractions of *E. coli* cells grown in standard laboratory conditions (Fig. S6). However, there was an increase in Lpp^ΔK58^ accumulation in the IM of cells grown under restrictive conditions (Fig. S6). LamB, an OM β-barrel protein (1), was restricted mostly to the OM fractions under these conditions (Fig. S6). Overall, these results alluded to a reduction in Lpp maturation flux in cells subjected to this environmental stress condition. A mild increase in Lpp^ΔK58^ retention in the IM fractions of the *dcrB* deletion gradient was seen, suggesting Lpp maturation is further reduced in the mutant background (Fig. S6). However, pro-Lpp^ΔK58^ accumulation could not be detected under the test conditions, presumably due to low levels of the precursor protein being diluted across the fractions (Fig. S6). Overall, the results lent support to the idea that the removal of Lpp-PGN links, while promoting viability of the *dcrB* null under stress, does not restore Lpp maturation to WT levels.

### DcrB’s role largely lies in facilitating Lpp maturation

To further probe a role for DcrB in Lpp maturation, we revisited the biogenesis properties of an Lpp mutant, Lpp^L10P^, identified and characterized many years ago (59). Lpp^L10P^ was reported to be more efficiently released from spheroplast membranes relative to WT Lpp (59). Structural predictions of Lpp^L10P^ reveal a trimeric α-helical coiled-coil, similar to WT Lpp, but with low binding confidence at its N-terminal end that bears the proline substitution (Fig. S7A). We reasoned that if Lpp^L10P^ was processed more efficiently at the IM, cells expressing this Lpp derivative would be less reliant on *dcrB* under stress. We conducted immunoblots of *lpp* or *dcrB lpp* cells expressing plasmid-borne Lpp^ΔK58^ or Lpp^L10P, ΔK58^ grown in the presence of sub-MIC Glb (0.25× MIC of WT) and probed for Lpp. As expected, the *dcrB* null cells revealed greater accumulation of pro-Lpp^ΔK58^ and prepro-Lpp^ΔK58^ forms relative to *dcrB*^+^ cells, reiterating a role for DcrB in promoting Lpp maturation under Glb stress (Fig. 4C). In contrast, pro-Lpp^L10P,ΔK58^ form accumulated to more or less similar amounts in *dcrB*^+/−^ cells, while the prepro-Lpp^L10P,ΔK58^ bands were barely detectable. These findings pointed to a diminished role for DcrB in the presence of an Lpp variant that is processed more efficiently.

To understand whether DcrB’s role in lipoprotein maturation extends beyond that of Lpp, we examined the maturation of RcsF, another model lipoprotein and a part of the well-studied Rcs extracytoplasmic stress response. The accumulation of pro-RcsF in *lpp* and *lpp*^ΔK58^ cells, with or without *dcrB*, in response to Glb (0.5× MIC of WT) stress was tested by immunoblotting for RcsF. No significant changes to the accumulation of pro-RcsF were observed in these strain backgrounds (Fig. S8).

In parallel, we also investigated Rcs activation in the *dcrB* mutant under stress. One among diverse inducing signals of the Rcs extracytoplasmic stress response is the accumulation of IM RcsF due to lipoprotein maturation defects (60). We examined a chromosomal *rprA-lacZ* fusion as a proxy for Rcs activation; *rprA* encodes a small RNA that is a well-studied member of the Rcs regulon (61). Deletion of *dcrB* showed a significant ~4-fold RcsF-dependent increase in Rcs activity relative to the WT (Fig. S9A and B). Additional deletion of *lpp* or the introduction of the *lpp*^ΔK58^ allele in the *dcrB* null downregulated Rcs response by similar amounts (Fig. S9B). As expected, elevated levels of LspA reduced Rcs activation in the *dcrB* mutant (Fig. S9C). Rcs levels were more or less similarly dampened in the *dcrB lpp* and *dcrB lpp*^ΔK58^ backgrounds, regardless of LspA overexpression (Fig. S9C). The basis for a bimodal Rcs response in *dcrB* null colonies is not clear (Fig. S9A). We speculate it reflects a positive autoregulatory feedback loop in the complex Rcs signaling cascade leading to two heritable states (61). In toto, these results indicate that RcsF maturation flux is not substantially impacted by DcrB.

### DcrB physically interacts with Lpp

**Fig 5:**
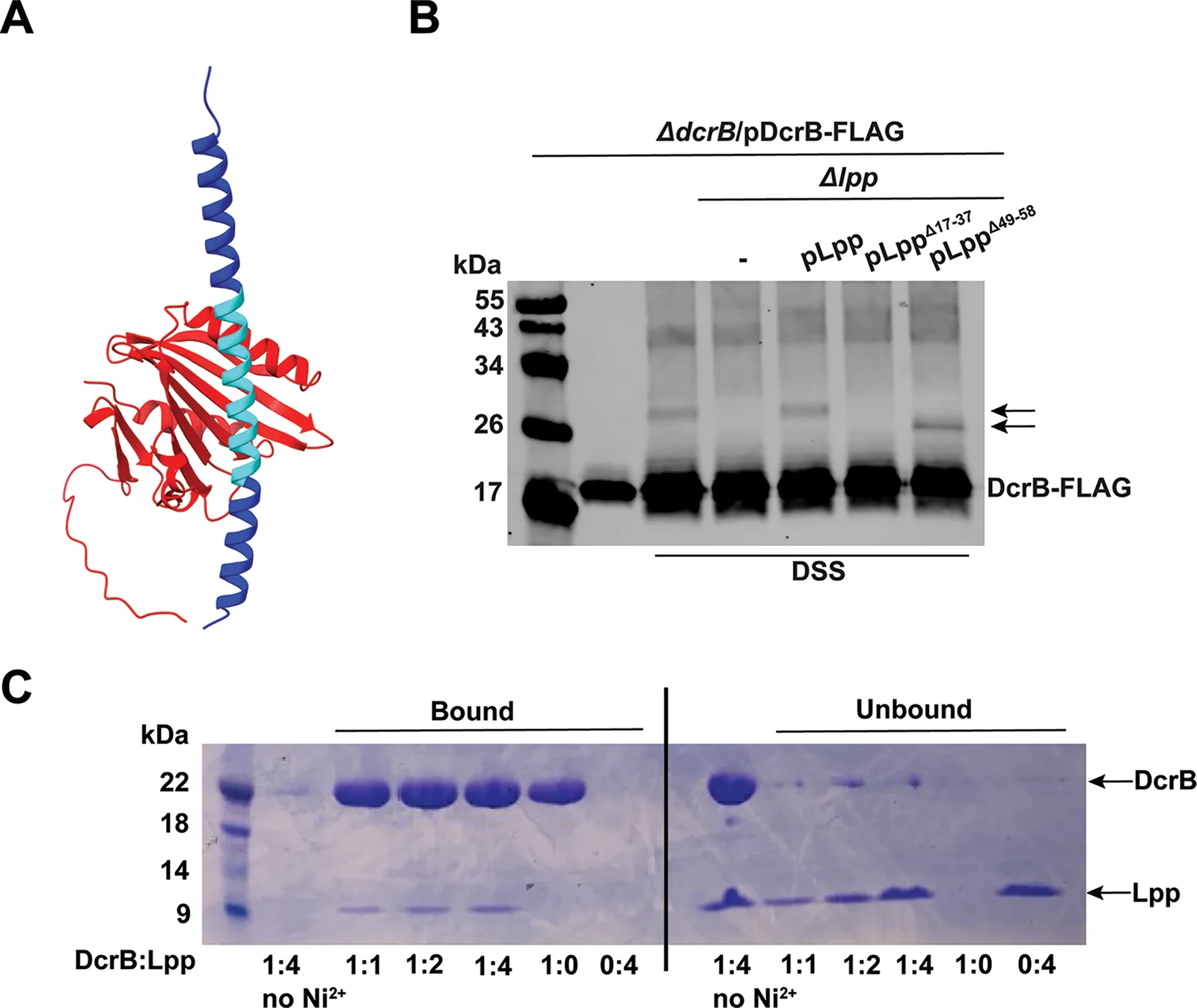
DcrB interacts with Lpp. (**A**) Structural model of DcrB/Lpp interaction predicted by AF3. DcrB and Lpp are colored in red and blue, respectively. The amino acid residues 17–37 of mature Lpp are shown in cyan. (**B**) Putative DcrB/Lpp complexes were examined by *in vivo* chemical crosslinking assays using a functional pBAD33-DcrB-FLAG fusion (pMRB58) in *dcrB* (AM135) or *dcrB lpp* (EL656) cells and where relevant also bearing pUC18-based Lpp (pMRB61), or Lpp^Δ17–37^ (pMRB63), or Lpp^Δ49–58^ (pMRB62) variants were assayed and detected using an anti-FLAG antibody as described in Materials and Methods. Because the Lpp^Δ17–37^ variant is reportedly not detected well by the Lpp antibody used in this study (36), twice the volume was loaded for this sample. The known position of DcrB-FLAG is indicated. The top arrow indicates the putative DcrB/Lpp monomer-monomer complex (~28 kDa) and the bottom one, a DcrB/Lpp^Δ49–58^ monomer-monomer complex (~26 kDa). (**C**) A purified cytoplasmic 10×His-FLAG-TEV-DcrB (pMB1) at 10 μM was coupled to Ni-NTA and used as bait for a tagless cytoplasmic Lpp (pMB2) that was added in varying amounts (0–40 μM). Control lanes with each protein (DcrB at 10 μM and Lpp at 40 μM), and one with both proteins but no beads are included, and show no detectable levels of bound Lpp. Unbound protein was collected, followed by elution of the bound protein from the Ni-NTA affinity column. Both unbound and bound fractions were separated on a 4–20% Bis-Tris Page gel and visualized using Coomassie blue staining. A representative gel image of at least three or more independent experiments is shown.

We wished to better understand how DcrB might promote Lpp maturation. One possibility was that DcrB interacts with Lpp and enhances Lpp’s transit through the IM. Structural modeling of the two proteins lent support for the DcrB/Lpp interaction. AlphaFold3 (AF3) predictions revealed DcrB/Lpp monomer/monomer interactions of very high confidence (Fig. 5A and Fig. S10). The interaction converged on the same sites in both proteins and in the same orientation with the N-terminal Cys residues positioned in the same place in the various predictions.

To investigate potential DcrB and Lpp interactions in the cell, we examined whether DcrB and Lpp are physically linked by conducting an *in vivo* chemical crosslinking assay essentially as described (36). To that end, we expressed a FLAG-tagged version of DcrB in *dcrB* and *dcrB lpp* mutants, treated them with the crosslinking agent disuccinimidyl suberate (DSS), and probed for DcrB by immunoblotting for FLAG. Of note, plasmid-borne expression of *dcrB-FLAG* restored viability of *dcrB* null in NA medium at the restrictive temperature, indicating the functionality of the fusion construct (Fig. S11A). In the absence of the crosslinking agent, we detected an intense band corresponding to monomeric DcrB (~20 kDa) (Fig. 5B). In the presence of the crosslinker, a higher-molecular-weight (~28 kDa) band was detected in *lpp*^+^ but not in *lpp* null cells, possibly representative of a DcrB/Lpp monomer-monomer complex (Fig. 5B).

To understand if the putative ~28 kDa band is a *bona fide* complex of DcrB/Lpp, we cloned and ectopically expressed WT *lpp*, an *lpp* mutant lacking amino acid residues 17–37 of the mature protein (*lpp*^Δ17–37^), and an *lpp* variant lacking the last heptad and carboxy-terminal residues of the mature protein (*lpp*^Δ49–58^), and repeated the crosslinking assays as above. AF3 predictions of DcrB/Lpp interactions converge on Lpp amino acid residues 17–37 of the mature protein as the putative interaction site of DcrB (Fig. S10). As expected, the band migrating at ~28 kDa reappeared upon complementation with WT Lpp but could not be detected in strains expressing Lpp variants (Fig. 5B and Fig. S11B). A faster-migrating band corresponding to the size of an apparent DcrB/Lpp^Δ49–58^ complex (~26 kDa) was detected upon expression of the Lpp mutant lacking the carboxyl-end residues (Fig. 5B). But a band representing DcrB/Lpp^Δ17–37^ complex was not detected (Fig. 5B). These findings corroborated the AF3-predicted binding confidence of each Lpp variant to DcrB (Fig. S10). Of note, in the presence of the crosslinker, an approximately 40 kDa band was present in all the lanes and could represent a DcrB dimer (Fig. 5B).

The crosslinking results suggested that Lpp^Δ17–37^ binds DcrB poorly. This implied that Lpp^Δ17–37^ accumulation would be similar regardless of the presence of *dcrB* in Glb-treated cells. Indeed, we found this to be the case: pro- and prepro-Lpp^Δ17–37,ΔK58^ amounts were mostly indistinguishable in *dcrB*^+/−^ cells (Fig. 4C). Together, we infer that DcrB and Lpp interact *in vivo*, potentially at a 1:1 stoichiometry, and that Lpp amino acid residues 17–37 likely represent the interaction site of DcrB.

We also examined the Lpp^L10P^ interaction with DcrB via *in vivo* crosslinking as above. AF3-predicted DcrB/Lpp^L10P^ interactions with high binding confidence (Fig. S10). Our experimental results validated the prediction as we detected a DcrB/Lpp^L10P^ complex of ~28 kDa, a size similar to that of DcrB/Lpp (Fig. S7B). This result suggested that while Lpp^L10P^ is less reliant on DcrB, presumably due to its efficient release from the IM, it can still interact with DcrB in the cell.

Finally, to examine whether Lpp and DcrB interact directly, we conducted a pull-down assay with purified proteins. Size exclusion chromatography of tagless Lpp revealed that the protein ran mostly as a trimer with a minor peak corresponding to the molecular weight of the monomer (Fig. S12A). 10×His-FLAG-TEV-DcrB revealed a primarily monomeric peak with a small fraction running at the molecular weight corresponding to a dimer (Fig. S12A). This is consistent with the observed less prominent ~40 kDa band in the crosslinking assays (Fig. 5B). The soluble His-FLAG-tagged DcrB coupled to nickel beads was used as a bait and could pull down tagless Lpp in a specific manner (Fig. 5C and Fig. S12B). Collectively, these results point to a physical association of DcrB and Lpp in the cell that is likely direct.

## DISCUSSION

Lpp biogenesis has been studied extensively over the years, and it has long been recognized that it is Lpp-mediated crosslinking to the PGN, not Lpp accumulation at the IM *per se*, that is toxic (41). Here, we describe the identification and characterization of a molecular safeguard for Lpp biogenesis mediated by DcrB, an IM lipoprotein of previously unknown function (43, 46, 62). Overall, our findings establish a critical role for DcrB in protecting cells from lipoprotein maturation stress through concomitant effects: enhancing the efficiency of Lpp maturation and alleviating IM Lpp-PGN crosslinking.

We found that deletion of *dcrB* sensitized cells to Glb and deletion of *lpp* was epistatic to the deletion of *dcrB* with respect to drug resistance, placing *lpp* downstream of *dcrB* in this pathway. *dcrB* null cells revealed increased accumulation of precursor Lpp forms under sub-MIC Glb exposure, pointing to reduced LspA processing capacity in the absence of DcrB. In contrast, precursor forms of a fast-release Lpp variant, Lpp^L10P^, accumulated to similar levels in *dcrB*^+/−^ cells, suggesting that when Lpp release is accelerated *dcrB* becomes dispensable. Furthermore, precursor forms of an Lpp variant, Lpp^Δ17–37^, which did not form a detectable complex with DcrB *in vivo*, were indistinguishable between *dcrB*^+/−^ cells. Together, these findings predict that DcrB is involved in enhancing Lpp maturation.

Independent of Glb stress, deletion of *dcrB* caused growth defects under low salt and low temperature conditions, which were suppressed by deletion of *lpp* or by the deletion of the PGN crosslinking residue of Lpp, K58. This implied a DcrB-dependent accumulation of aberrant IM-Lpp-PGN crosslinks and is consistent with impaired precursor biogenesis leading to a population of Lpp molecules establishing inappropriate IM-PGN crosslinks. Multicopy Lgt or Lnt each provided mild rescue to the *dcrB* null under these conditions, while multicopy LolCDE did not. However, increasing the pool of LspA restored robust growth to the *dcrB* null, localizing the defect to LspA-mediated cleavage, the rate-limiting step in the lipoprotein maturation pathway (11). As the levels of LspA were essentially similar between WT and *dcrB* null in restrictive growth conditions, these results offer support that the constraint in the absence of *dcrB* is LspA processing efficiency. The maturation of RcsF, another well-studied lipoprotein, was not significantly influenced by DcrB in *lpp* null or *lpp*^ΔK58^ backgrounds; in the latter, there remains competition for shared lipoprotein maturation pathway capacity. The residual Rcs activation observed in the *dcrB* null upon removal of *lpp*, or unhooking Lpp from the PGN, or increased expression of LspA, probably stems from other aspects of RcsF-mediated envelope disorder signals rather than those from RcsF maturation defects.

How DcrB, itself an IM lipoprotein, promotes Lpp maturation and reduces the formation of incorrect Lpp-PGN linkages remains to be determined. Our results converge on a model in which DcrB engages with precursor Lpp, likely around amino acid residues 17–37 (numbers corresponding to the mature protein) interface, and that this engagement facilitates the Lpp precursor to be acted upon by LspA. This model is supported by structural predictions (AF3) reporting a DcrB/Lpp monomer-monomer interaction with high confidence, *in vivo* chemical crosslinking revealing a DcrB/Lpp complex, which is not detectable upon deletion of Lpp residues 17–37, and pull-down data demonstrating a physical interaction between purified DcrB and Lpp. We posit that in the absence of DcrB, a fraction of the Lpp molecules lingering at the IM form inappropriate self-associations. However, Lpp oligomerization at the IM is considered to be a rare event (59, 63, 64). Under standard growth conditions, the efficient processing of lipoproteins coupled with the very slow kinetics of Lpp trimerization ensures that this is the outcome (59, 63, 64). Nevertheless, when lipoprotein maturation rates are reduced, and an abundant cargo such as Lpp accumulates in the IM, it is unclear whether premature oligomerization is a plausible fate for a subset of Lpp molecules. Given that the unfolding of Lpp trimers is exceptionally slow (63, 64) and that the lipoprotein modification and transport enzymes can accommodate only one lipoprotein molecule in each cycle (52, 65–68), such Lpp oligomers would fail to be modified and released from the IM and in essence face a “dead-end” in their journey to the OM. Earlier studies testing Lpp release in spheroplasts revealed that in the absence of LolA, a portion of Lpp presumably trimerizes in the IM and that this trimeric Lpp is unable to engage with the Lol transporter and is not released from the membrane (17, 59). Indeed, Lpp^L10P^ was identified as a variant that is released more efficiently from spheroplasts in the same study (59). While AF3 predicts Lpp^L10P^ can form trimers, the confidence scores are low at the N-terminus bearing the proline substitution. The folding/unfolding kinetics of the predicted Lpp^L10P^ associations are unknown; therefore, whether the oligomeric state of Lpp^L10P^ underlies the enhanced processing and reduced reliance on DcrB at the IM is not known.

How might IM-stalled Lpp precursors come to form more crosslinks to the PGN? One possibility is that the DcrB/Lpp interaction may normally interfere with crosslinking by the LdtB transpeptidase (or promote LdtF-mediated removal of crosslinks), such that in DcrB’s absence, the lingering Lpp precursors are crosslinked more efficiently. However, this alone is insufficient to explain the enhanced IM retention of the PGN-untethered Lpp^ΔK58^ variant in the *dcrB* null. Instead, our data support a model in which DcrB accelerates Lpp’s transit through the LspA processing step, thereby reducing the formation of toxic IM-Lpp-PGN crosslinks. Without DcrB, we propose that a subset of the lingering Lpp precursors form premature self-associations, which may in turn influence their crosslinking to the PGN. This would not be unprecedented, as a recent study implicates Lpp oligomerization in PGN crosslinking (69).

Although we favor the above model, an alternative possibility is that DcrB acts on LspA and preserves the shared pathway capacity of this enzyme. However, it is unlikely that DcrB acts as a dedicated LspA factor based on the following observations: (i) the DcrB-dependent growth patterns of IM-Lpp variants, which cannot be lipidated by Lgt and are therefore not LspA substrates, (ii) the minimal impact of DcrB on the transit of Lpp^Δ17–37^, which DcrB likely binds poorly, compared to its impact on WT Lpp transit, and (iii) the limited influence of DcrB on RcsF maturation when Lpp is present but untethered from the PGN. Instead, the data support Lpp recognition by DcrB. Our results cannot, however, exclude a broader role for DcrB in lipoprotein maturation by influencing the processing of other lipoproteins.

The functional region of DcrB encompasses a DUF1795 domain; in *M. tuberculosis* and *P. aeruginosa*, the DUF1795-containing proteins LpqN and EagT6, respectively, bind hydrophobic ligands that participate in trans-envelope pathways (44, 45). Indeed, EagT6 is a chaperone involved in effector delivery via the type VI secretion system (45). Based on the structural similarity of DcrB to these proteins and the results from the present study, it is tempting to propose an analogous “chaperone-like” role for DcrB, wherein DcrB binds the hydrophobic face of the helical domain of Lpp (around aa residues 17–37) and promotes its biogenesis across the *E. coli* cell envelope. The cellular levels of DcrB are reported to be several orders of magnitude lower than those of the highly abundant Lpp (29), a disparity that is consistent with such a role. Ongoing efforts in the laboratory are expected to uncover mechanistic details underlying the DcrB/Lpp interaction in *E. coli*.

## MATERIALS AND METHODS

### Strains, plasmids, and growth conditions

Strains used in this study are isogenic derivatives of *E. coli* K12 MG1655 unless specified otherwise. All strains, plasmids, and primers used in the study are reported in Tables S2 to S4. Plasmids were constructed using standard molecular techniques. Additional details of plasmid construction, where applicable, are described in the Supplemental material. PCR amplification was performed using Phusion Flash polymerase (ThermoFisher) using MG1655 chromosomal DNA as the template, unless mentioned otherwise. Plasmid DNA and PCR fragments were purified using the Zyppy miniprep kit (Zymo Research) or the PCR clean-up kit (Macherey-Nagel), respectively. Plasmid clones and PCR products were verified by DNA sequencing (Azenta). Null mutations were derived from the Keio collection (70) and introduced into appropriate strains by P1vir transduction; when necessary, the Kan^R^ markers were excised via FLP recombination as previously described (71). Strains were grown in either LB medium (1% Tryptone, 0.5% Yeast extract, and 1% NaCl), LB medium without the addition of NaCl (LB [0% NaCl]), Nutrient agar/broth (NA and NB, respectively), or 2× yeast extract tryptone (YT) medium routinely at 37°C or at temperatures specified in the figures and/or figure legends. When necessary, the medium was supplemented with antibiotics at concentrations of 100 μg/mL ampicillin (Amp), 20 μg/mL chloramphenicol (Cm), or 50 μg/mL kanamycin (Kan). For induction of plasmid-based factors, growth medium was supplemented with L-arabinose (L-Ara) and isopropyl β-d-1-thiogalactopyranoside (IPTG) at final concentrations reported below or in the figures or figure legends.

### Efficiency of plating assays

Cultures were grown overnight under permissive conditions in LB (1% NaCl) containing antibiotics, when necessary, at 37°C. Overnight cultures were washed three times in NB or LB (0% NaCl), normalized to OD_600_ of 1, and 10-fold serially diluted (vol/vol) into appropriate media. An aliquot of 4 μL of each dilution was spotted on appropriate media, and plates were incubated for 16–18 h at 30°C or 37°C and ~60 h at 20°C. Plates were imaged using a Gel Imager (Syngene).

### Minimal inhibitory concentration (MIC) determination

MIC for strains treated with Glb was determined essentially as described (72). Briefly, overnight cultures were diluted in 96-well microtiter plates to a final OD_600_ of ~0.001 in LB (1% NaCl) and grown for 16 h at 37°C in a Spectramax M2 (Molecular Devices) plate reader with varying concentrations of Glb. The reported MIC corresponds to the lowest Glb concentration that prevented any visible cell growth.

### Microscopy

Overnight cultures grown in LB (1% NaCl) at 37°C were washed and subcultured 1:100 (OD_600_ = 0.03) into LB (0% NaCl) or NB and grown for 14–16 h at 20°C until OD_600_ of 0.4–0.5 or 18–24 h until OD_600_ of 0.3–0.4, respectively, at which point, 4 μL of each strain was spotted onto a 2% agarose pad for visualization by phase microscopy with a Nikon TiE microscope with a charged-coupled device (CCD) camera essentially as described (46).

Plasmolysis bay cytological assays were performed as described in reference 49 with some modifications. Overnight cultures of TB28 strains bearing mScarlet-I fusion plasmids (pDSW204-Lpp-mScar, pDSW204-Lpp^C21G^-mScar, or pDSW204-OppB_TM_-Lpp-mScar) were subcultured to OD_600_ of 0.05 in LB (1% NaCl) with Amp and grown until OD_600_ ~0.5–0.6 with no IPTG added. At this point, 0.5 mL was harvested, centrifuged at 6,000 rpm for 5 min, and the pellet was resuspended in either 10 μL of LB (1% NaCl) as a control, or 10 μL of plasmolysis solution (15% sucrose, 25 mM HEPES, pH 7.4, and 20 mM NaN_3_). Within 15 min of plasmolysis, cells were immobilized on 1% agarose pad without or with 15% sucrose (to maintain plasmolysis), and localization of the fusion protein was imaged via phase and fluorescence (TxRED filter) microscopy as described above. Localization of pDSW206-DcrB-mScar or pDSW206-DcrB_OM_-mScar was analyzed similarly as described above, with the exception that inducer IPTG was added to a final concentration of 100 μM from the beginning of the subculture.

### Immunoblot analyses

Protein extraction was performed using BugBuster (MilliporeSigma) unless otherwise specified. Briefly, cells were grown to OD_600_ = 0.5–0.6, normalized to OD_600_, washed three times in ice-cold 0.2 M Tris-HCl (pH 8.0), and spun for 10 min at 16,100 × *g* at 4°C. Next, cell pellets were suspended in BugBuster (diluted in 100 mM Tris-HCl, pH 8.0, containing 100 mM NaCl) with 50 U/mL Benzonase nuclease (MilliporeSigma) with or without 1 KU fresh rLysozyme (MilliporeSigma). The cell suspensions were lysed by gentle shaking for 45 min at room temperature and then centrifuged at 16,100 × *g* for 15 min at 4°C to pellet the cell debris. Supernatants containing protein extracts were mixed with an equivalent volume of 2× Tricine-SDS sample buffer with 4% BME, heated to 85°C for 3 min, and resolved by electrophoresis in 16% Tricine (Novex) gels at 125 V for 3–4 h. Proteins were transferred to 0.22 µm nitrocellulose membranes for immunoblotting. Membranes were briefly stained for total protein using the BLOT-FastStain (GBiosciences), as per the manufacturer’s instructions. Membranes were then blocked using PBS-T (phosphate-buffered saline with 1% Tween-20) containing 5% milk for 1 h at room temperature and probed with an anti-Lpp rabbit polyclonal antibody at 1:500,000 at 4°C overnight with gentle shaking. This was followed by incubating the membrane in an IR-conjugated goat anti-rabbit secondary antibody at 1:10,000 (LI-COR) for 1 h at room temperature. Bands were visualized using the LI-COR Odyssey Clx imager and quantified using ImageQuant Studio (LI-COR).

### Structural analysis

AlphaFold3 predictions were obtained with AlphaFold version 3 (73) using the AlphaFold server (https://alphafoldserver.com). ChimeraX (74) was used to visualize the structural models and generate images.

### Crosslinking assays

*In vivo* chemical crosslinking of DcrB-FLAG fusion and Lpp was done essentially as described (36). Briefly, overnight cultures of strains bearing pBAD33-DcrB-FLAG fusion and pUC18-Lpp or pUC18-Lpp variants grown in LB (1% NaCl with 0.2% glucose) were washed and subcultured into fresh medium supplemented with 0.02% L-Ara and Cm at 20 μg/mL, and where appropriate additionally supplemented with 5 μM IPTG and 100 μg/mL Amp for induction of Lpp, at OD_600_ ~0.2 and grown to an OD_600_ ~0.5–0.7. At this point, cells were normalized by OD_600_ and concentrated to an OD_600_ ~10 in a final resuspension in 125 μL ice-cold PBS. The cells were washed three times at 16,100 × *g* in 1 mL ice-cold PBS and resuspended in 125 μL ice-cold PBS. The crosslinking agent DSS was added to each tube at 1,000 μg/mL final concentration and tubes were incubated for 30 min at room temperature. The reactions were quenched by addition of Tris-HCl (pH 7.4) at 250 mM final concentration, incubated at room temperature for an additional 15 min, washed in ice-cold PBS, and resuspended in 10 mM Tris-HCl (pH 7.4) containing 4× Laemmli SDS reducing buffer (ThermoFisher) to a final OD_600_ ~2.5. Samples were heated to 55°C for 10 min, centrifuged to pellet cell debris, and supernatants were resolved by electrophoresis in 16% Tris-glycine (Novex) gels at 125 V for 2–3 h. Proteins were transferred to 0.22 µm nitrocellulose membranes and immunoblot analysis was conducted as above. For detection of FLAG, membranes were probed with M2 monoclonal anti-FLAG mouse antibody (Sigma) at 1:1,000 and IR-conjugated goat anti-mouse secondary antibody at 1:20,000 dilution.

### Protein purification

*E. coli* BL21-pLysS carrying the pNYCOMPS-N-term-DcrB plasmid was used to overexpress an N-terminal 10×His-FLAG-TEV tagged cytoplasmic DcrB variant (residues 21–185 of the full-length protein). Cells were grown in 2× YT medium at 37°C to OD_600_ ~1, at which point IPTG at 1 mM final concentration was added, and the cells were grown for an additional 4 h at 37°C. At this point, cells were centrifuged, and the pellets were resuspended in lysis buffer (20 mM HEPES [pH 7.0], 150 mM NaCl, 40 mM imidazole, 0.5 mM TCEP, 1.2 μg/mL AEBSF, and 1 µg/µL DNase) using 25 mL buffer per 8 g of cells and disrupted by sonication. Cell debris was pelleted by centrifugation at 50,000 × *g* for 30 min at 4°C. The supernatant was added to 0.5 mL of Ni-NTA beads and incubated overnight at 4°C. The protein-beads mixture was collected in a gravity column and washed once with 30 mL of wash buffer (20 mM HEPES [pH 7], 150 mM NaCl, 70 mM imidazole, and 0.1 mM TCEP) before being eluted in 500 µL increments with elution buffer (20 mM HEPES [pH 7], 150 mM NaCl, 500 mM imidazole, and 0.1 mM TCEP). *A*_280_ measurement was taken for each eluted fraction, and the protein-containing fractions were pooled and dialyzed in 2 L of dialysis buffer (20 mM HEPES [pH 7.0], 150 mM NaCl, and 0.1 mM TCEP) overnight at 4°C. Protein was collected, concentrated to ~1 mL using a centrifugal concentrator (EMD Millipore), and run through size exclusion (Superdex S75 Increase 10/300; GE Healthcare). The purified protein was pooled and concentrated to ~1 mL. *A*_280_ measurement was recorded; the protein concentration was calculated using 1 Abs = 1 mg/mL, and the protein was aliquoted into 100 µL fractions before being stored at −80°C for further use.

*E. coli* T7 Express (NEB) carrying the pMCSG9-Lpp was used to overexpress an N-terminal 6×His-MBP-TEV-tagged cytoplasmic version of Lpp (amino acid residues 22–78 of the full-length protein). Cells were grown in 2× YT medium at 37°C to an OD_600_ of ~1. At this point, IPTG at 1 mM final concentration was added for plasmid induction, and the cells were grown for an additional 4 h at 37°C. Cells were pelleted, and the purification process was followed as described above for DcrB, with the exception of cleavage of the 6×His-MBP-TEV tag from Lpp by adding TEV protease (SIGMA) at a ratio of 1 unit of TEV protease per 10 units of protein during the dialysis step. After dialysis, the cleaved Lpp was separated from the tag by passing the sample three times through a gravity column containing 0.5 mL of washed Ni-NTA beads. The protein was then concentrated and run through gel filtration as described for DcrB purification above. Purified Lpp was collected and concentrated to ~300 µL, and the *A*_280_ was measured by NanoDrop spectrophotometer (ThermoFisher) using the reported extinction coefficient of Lpp (1490) in ExPASy. Purified protein was stored at −80°C for later use.

### *In vitro* DcrB-Lpp interaction

A cytoplasmic version of DcrB with an N-terminal 10×His-FLAG-TEV tag and a cytoplasmic, tag-free version of Lpp purified as described above were used in this assay. 10×His-FLAG-TEV-DcrB (100 µM) was coupled to 25 µL of Ni-NTA beads overnight at 4°C, then centrifuged at 1,000 × *g* for 5 min, and washed with 100 µL of wash buffer (20 mM HEPES [pH 7], 150 mM NaCl). Ten micromolar DcrB fusion bound to Ni was then combined in 1.5 mL microcentrifuge tubes with varying amounts of Lpp, ranging from 10 to 40 µM, with a total assay volume of 80 µL. Controls of no Lpp added to the bound DcrB, 40 µM of Lpp added to Ni-NTA beads only, and 10 µM of uncoupled DcrB mixed with 40 µM of Lpp were also included. DcrB/Lpp reactions were incubated at room temperature for 30 min with frequent, gentle shaking. Samples were then centrifuged at 1,000 × *g* for 5 min, and the supernatant was gently pipetted off as the unbound fraction. Pellets were then washed twice with 100 µL of wash buffer before the protein was eluted off the beads with 40 µL of elution buffer (20 mM HEPES [pH 7.0], 150 mM NaCl, and 500 mM imidazole) by incubating at room temperature for 20 min, then centrifuging for 5 min at 14,100 × *g*. The supernatant was collected as the bound fraction. Both bound and unbound fractions were separated by SurePAGE 4–20% Bis-Tris gel (GenScript) and visualized with Coomassie Blue stain.

## SUPPLEMENTAL MATERIAL

Supplemental material mbio.01845-26-s0001.pdf Fig. S1–S12, Tables S1–S4, and additional methods.

ASM does not own the copyrights to Supplemental Material that may be linked to, or accessed through, an article. The authors have granted ASM a non-exclusive, world-wide license to publish the Supplemental Material files. Please contact the corresponding author directly for reuse.
