# Supplementary material for: A molecular safeguard in Escherichia coli Braun’s lipoprotein (Lpp) biogenesis
Source: mBio. 2026 Sep 17;17(10):e01845-26. doi: 10.1128/mbio.01845-26 (PMC13712963; doi:10.1128/mbio.01845-26)
Supplement: Supplementary file 1 [file mbio.01845-26-s0001.pdf]

**Supporting information for**

# A Molecular Safeguard in *Escherichia coli* Braun's Lipoprotein (Lpp) biogenesis

Emily J. LaMarre[1,2,*], Makayla R. Braunlin[1,2,*], Brian Kloss[3], Faiza Rafi[1], Bogdan I. Iorga[4], and Anuradha Janakiraman[1,2,#]

[1] Department of Biology, 160 Convent Avenue, MR 526, The City College of CUNY, New York, NY, 100031, USA

[2] Program in Biology, The Graduate Center, CUNY, Fifth Avenue, New York, NY, 10016, USA

[3] New York Structural Biology Center, 89 Convent Avenue, New York, NY 10031, USA

[4] Université Paris-Saclay, CNRS UPR 2301, Institut de Chimie des Substances Naturelles (ICSN), 1 avenue de la Terrasse, Bât. 27, 91190 Gif-sur-Yvette, France

*equal contribution

#To whom correspondence should be addressed

Anuradha Janakiraman

Email: anuj@ccny.cuny.edu

**This PDF file includes:**

Figures S1 to S12
Supporting text
Tables S1 to S4
SI References

**FIGURE S1**

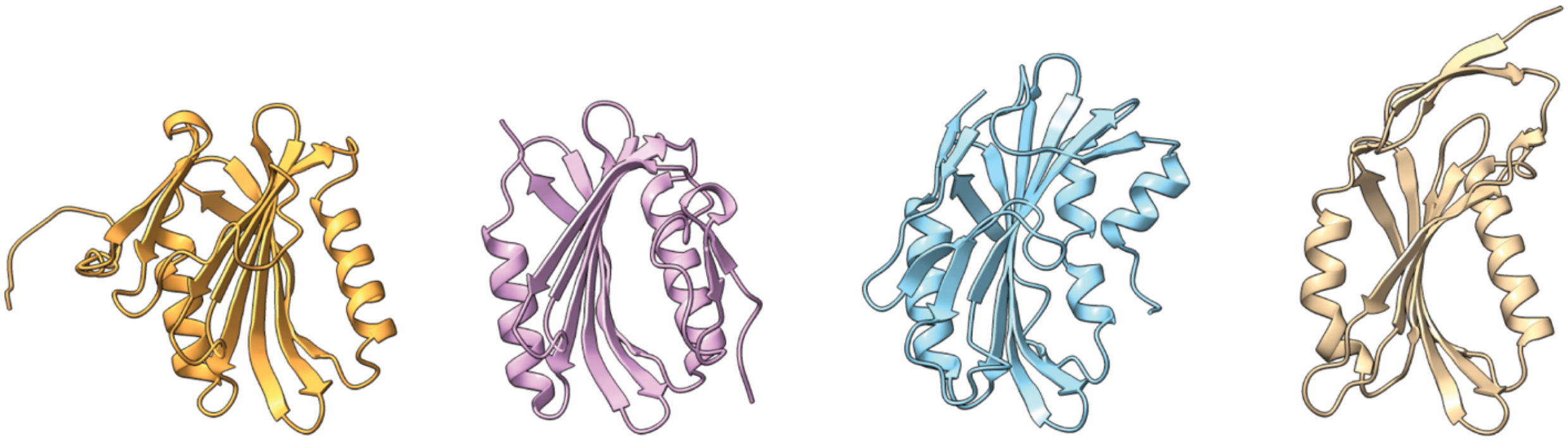


**Fig. S1. Structural homologs of *E. coli* DcrB.** The Z score matches to the AlphaFold predicted structure of *E. coli* DcrB are 16.7 (PDB:6E8A), 10.5 (PDB:6E5D), and 11.2 (PDB:1TU1), respectively.

## FIGURE S2

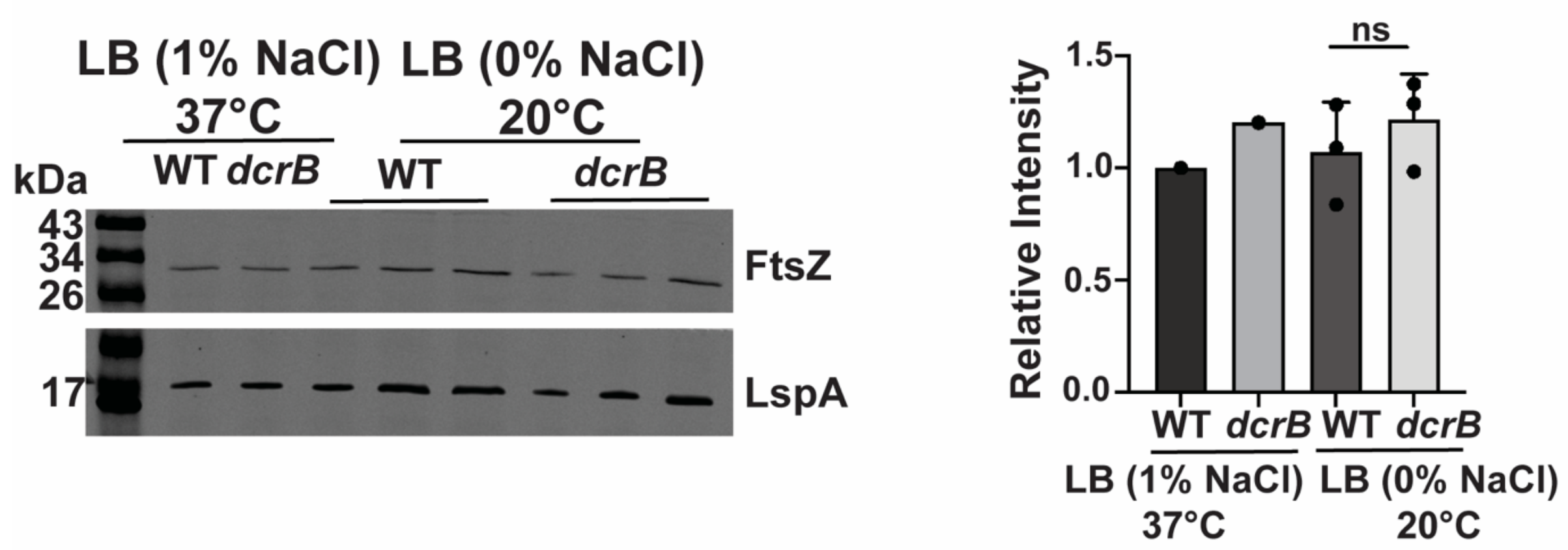


**Fig. S2. LspA levels are comparable between WT and *dcrB* null grown under non-permissive conditions.** A representative western blot of WT and isogenic *dcrB* null bearing an *lspA*-FLAG fusion (MB270, MB271), integrated into the native site on the chromosome, is shown. Cells were grown in LB (1% NaCl) at 37°C or LB (0% NaCl) at 20°C until mid-log. Proteins were harvested by normalizing to $OD_{600}$, resolved in 12.5% SDS-PAGE gel, and LspA levels were probed with an anti-FLAG antibody as described in Methods of the main text. The levels of FtsZ (~37 kDa) served as a loading and transfer control. The quantification of the relative intensities of the bands is reported on the right. The mean intensities with standard deviations of mutant relative to WT under standard condition are calculated based on 3 independent experiments. Statistical analysis was conducted using an unpaired t-test with p value = 0.453 marked as ns = not significant.

FIGURE S3

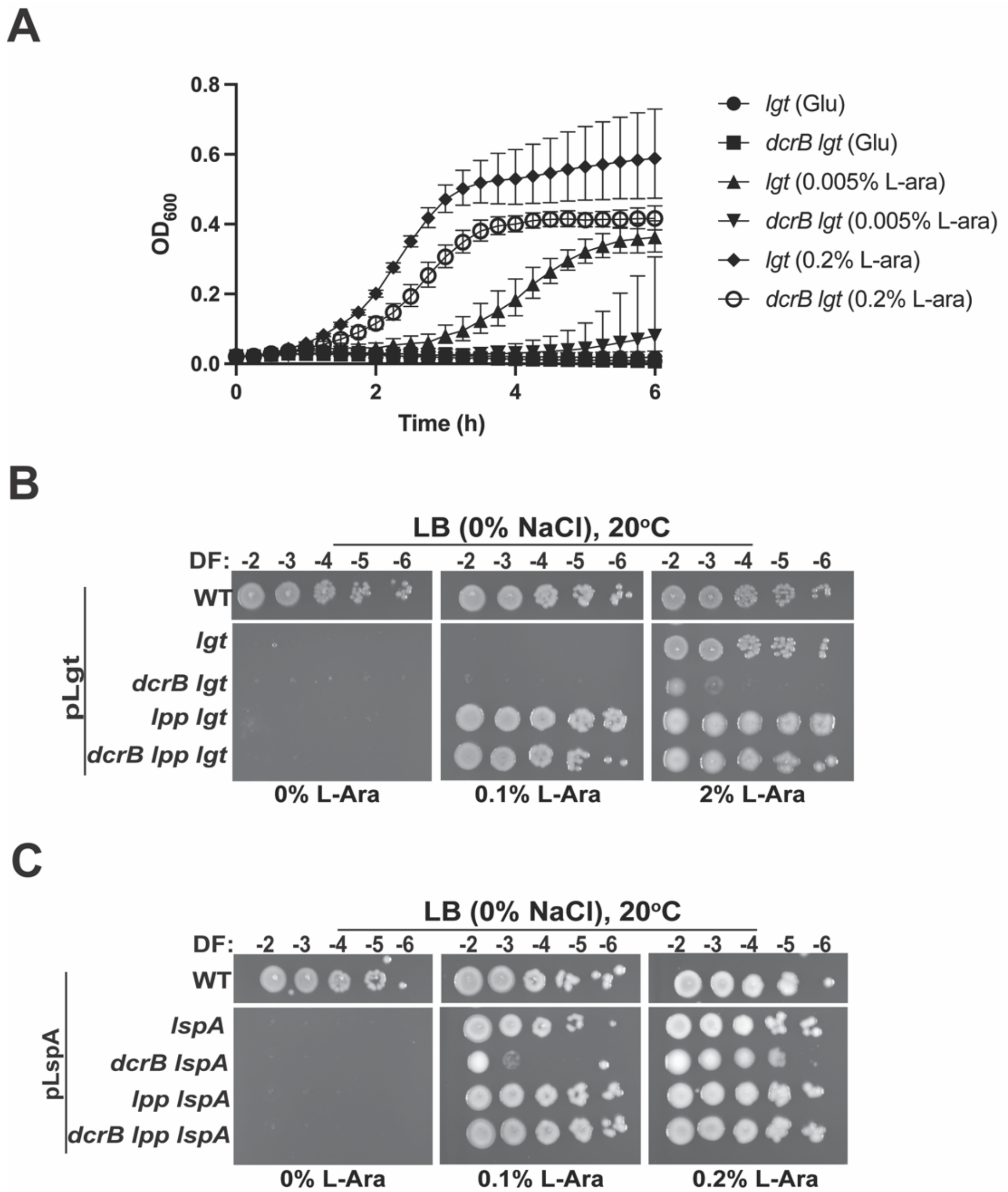


**Fig. S3. DcrB promotes *E. coli* growth under lipoprotein maturation stress conditions. (A)** Growth curves of *lgt* depletion strains in the presence (EL632) or absence of *dcrB* (EL634) are shown. Cells were grown in LB (1% NaCl) with L-Ara (Lgt+) overnight at 37°C and subcultured the next day into LB (1% NaCl) containing 0.2% glucose (Lgt-depletion), or 0.005% L-Ara, or 0.2% L-Ara (Lgt+) and growth monitored. The reported standard deviations are from 3 biologically independent repeats. **(B)** Overnight cultures of WT and isogenic *lgt* mutant bearing pCHAP9231-*lgt* plasmid (EL627, EL632) with or without additional deletions in *dcrB* (EL634), *lpp* (EL668), or

both (EL670), were normalized to $OD_{600}$ and aliquots of serial dilutions were spotted on LB (0% NaCl) plates containing indicated amounts of L-Ara and incubated for 60 h at 20°C prior to imaging. **(C)** Overnight cultures of WT (EL1231) and isogenic *lspA* mutant (EL1242) bearing pBAD33-*lspA* plasmid (pEL01) with or without additional deletions in *dcrB* (EL1244), *lpp* (EL1247), or *dcrB lpp* (EL1248) were treated as in (B). Representative plate images are shown.

**FIGURE S4**

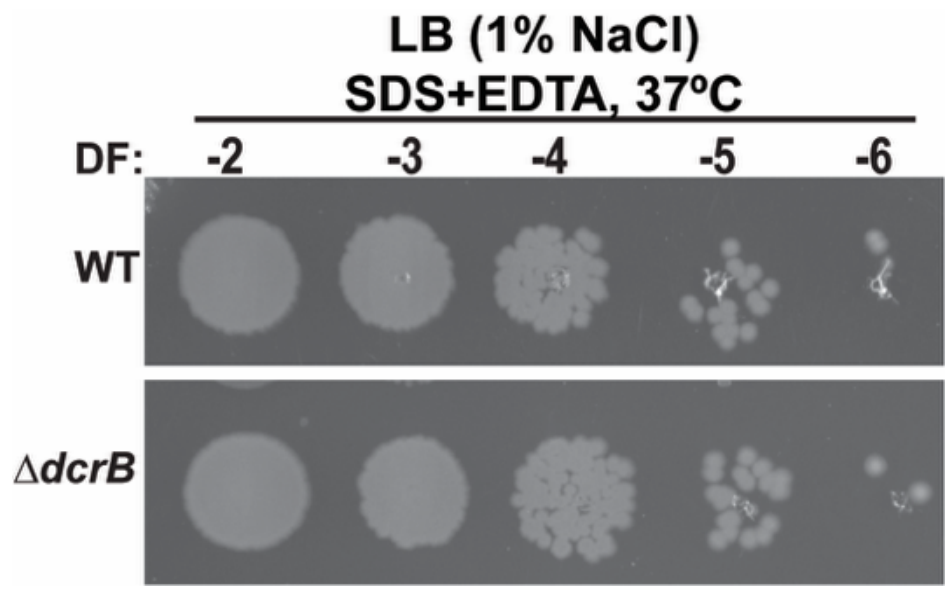


**Fig. S4. The permeability of the OM is not significantly altered in a *dcrB* null.** Overnight cultures of WT and isogenic *dcrB* deletion (AM135) grown in LB (1% NaCl) at 37°C were normalized to $OD_{600}$ and aliquots of serial dilutions were spotted on LB (1% NaCl) plates supplemented with 0.5% SDS and 0.8 mM EDTA and incubated for 16 h at 37°C prior to imaging. A representative plate image is shown.

## FIGURE S5

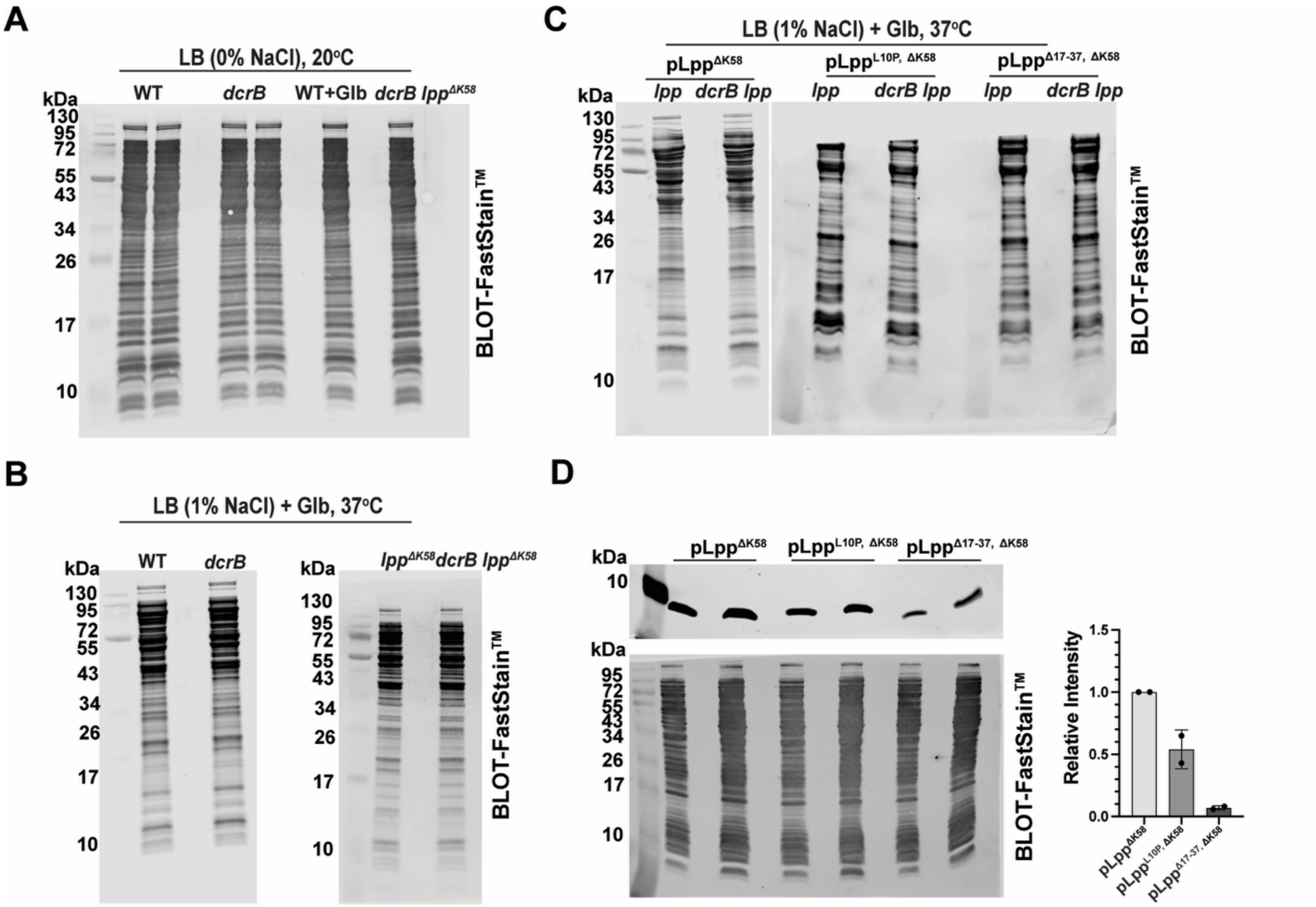


**Fig. S5. Total protein level controls. (A-C)** Membranes immunoblotted for Lpp or Lpp variants shown in **Fig. 4A-C** of the main text were stained for total protein using BLOT-FastStain™ (GBiosciences) and served as loading and transfer controls. **(D)** Immunoblots of plasmid-borne $Lpp^{\Delta K58}$ (pEL37), $Lpp^{L10P,\ \Delta K58}$ (pEL39), or $Lpp^{\Delta 17\text{-}37,\ \Delta K58}$ (pEL41) variants used in Δ*lpp* background in **Fig. 4C** of the main text. Cells were grown in the presence of 100 μM IPTG from the start of the subculture. Proteins were harvested by normalizing to $OD_{600}$, separated in a 16% Tris-glycine gel (Novex™) by electrophoresis, and immunoblotted with an anti-Lpp antibody as described in the Methods of the main text. Total protein staining served as a loading and transfer control. A representative image of two independent experiments is shown. The quantification of the relative intensities of the bands normalized to the total protein is reported on the right.

**FIGURE S6**

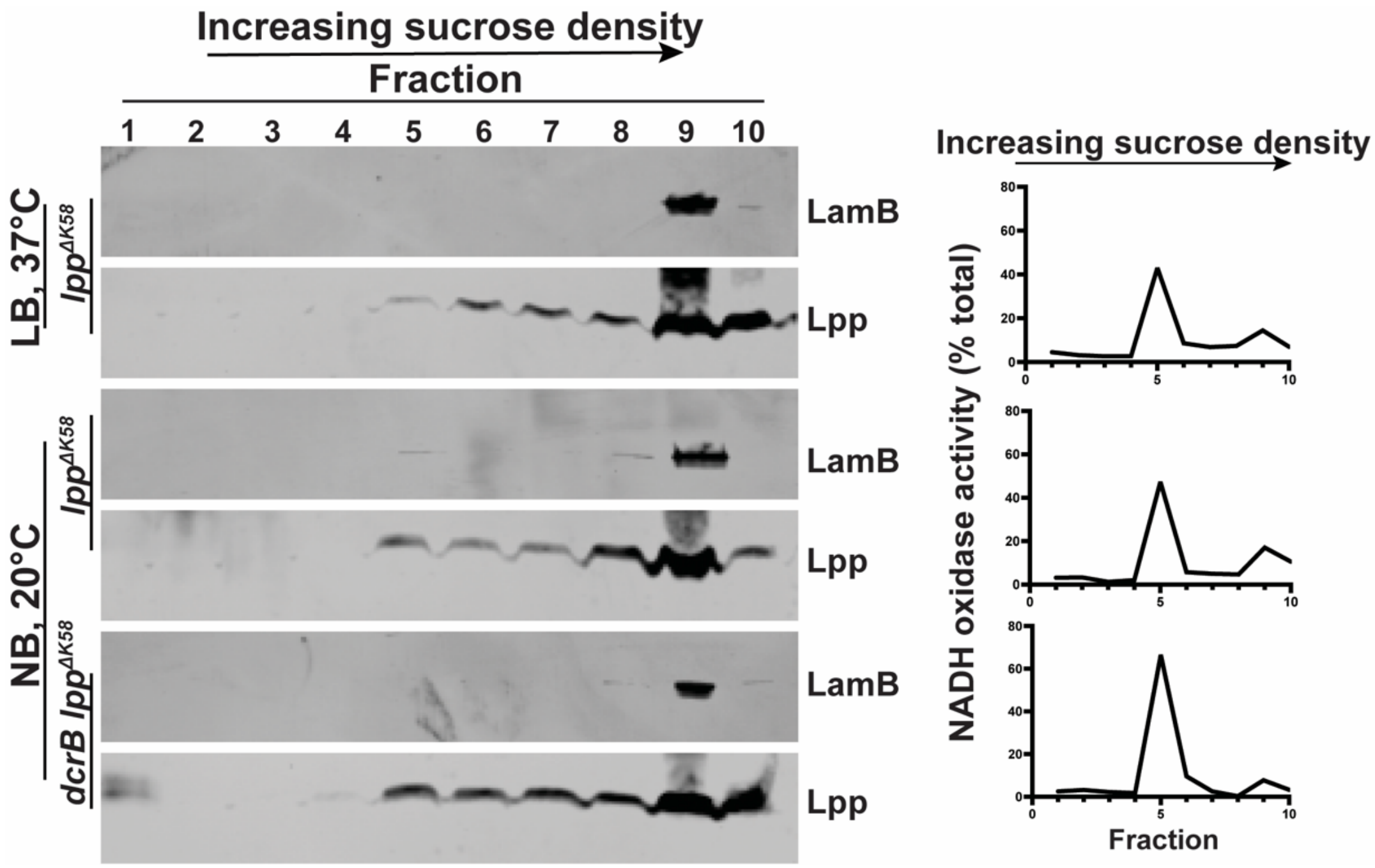


**Fig. S6. Results from membrane fractionation of *lpp*$^{\Delta K58}$ cells in *dcrB* +/- backgrounds grown in low salt and low temperature stress.** Inner and outer membranes were separated and fractions collected. Total proteins in each fraction from gradients of *lpp*$^{\Delta K58}$ (AM1712) cells grown under standard [LB (1% NaCl), 37°C] and restrictive conditions (NB, 20°C), and the mutant derivative *dcrB lpp*$^{\Delta K58}$ (AM1714) grown under restrictive conditions, were separated by 16% Tris-glycine (Novex$^{TM}$), and immunoblotted for Lpp$^{\Delta K58}$ and an OM β-barrel protein LamB. Fractions were analyzed for NADH oxidase activity, as an IM marker, and activity reported as a percentage of total activity. NADH oxidase activity peaked in fraction 5 in each strain under the conditions tested. See SI Methods for additional details.

**FIGURE S7**

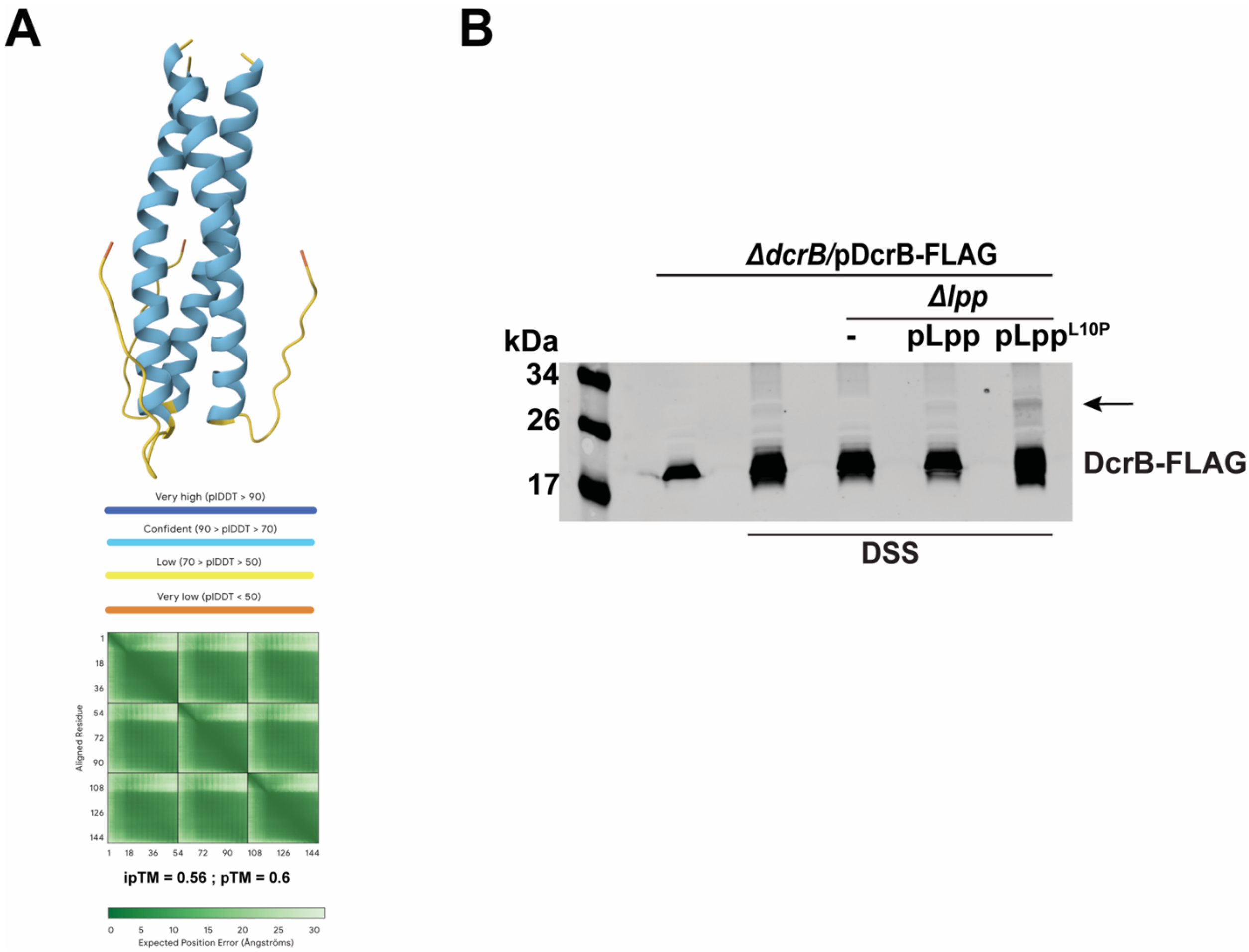


**Fig. S7. Lpp$^{L10P}$ AF3 prediction and *in vivo* interaction. (A)** AlphaFold predicted structure of Lpp$^{L10P}$ trimer. Predicted aligned error (PAE) plot is shown in green. The values of predicted template modelling (pTM) and interface predicted template modelling (ipTM) scores are also provided for the prediction. **(B)** Putative DcrB/Lpp$^{L10P}$ complex visualized by *in vivo* crosslinking assays using a functional pBAD33-DcrB-FLAG fusion (pMRB58) in *dcrB* (AM135) or *dcrB lpp* (AM814) cells and where relevant expressing either a pUC18-based Lpp (pMRB61) or Lpp$^{L10P}$ (pEL40) plasmid. Cells were grown, processed, and detected using an anti-FLAG antibody as described in the Methods of the main text. Lpp$^{L10P}$ was loaded at 4X sample volume relative to Lpp. The known position of DcrB-FLAG is indicated. The arrow indicates the putative DcrB/Lpp$^{L10P}$ monomer-monomer complex (~28 kDa).

**FIGURE S8**

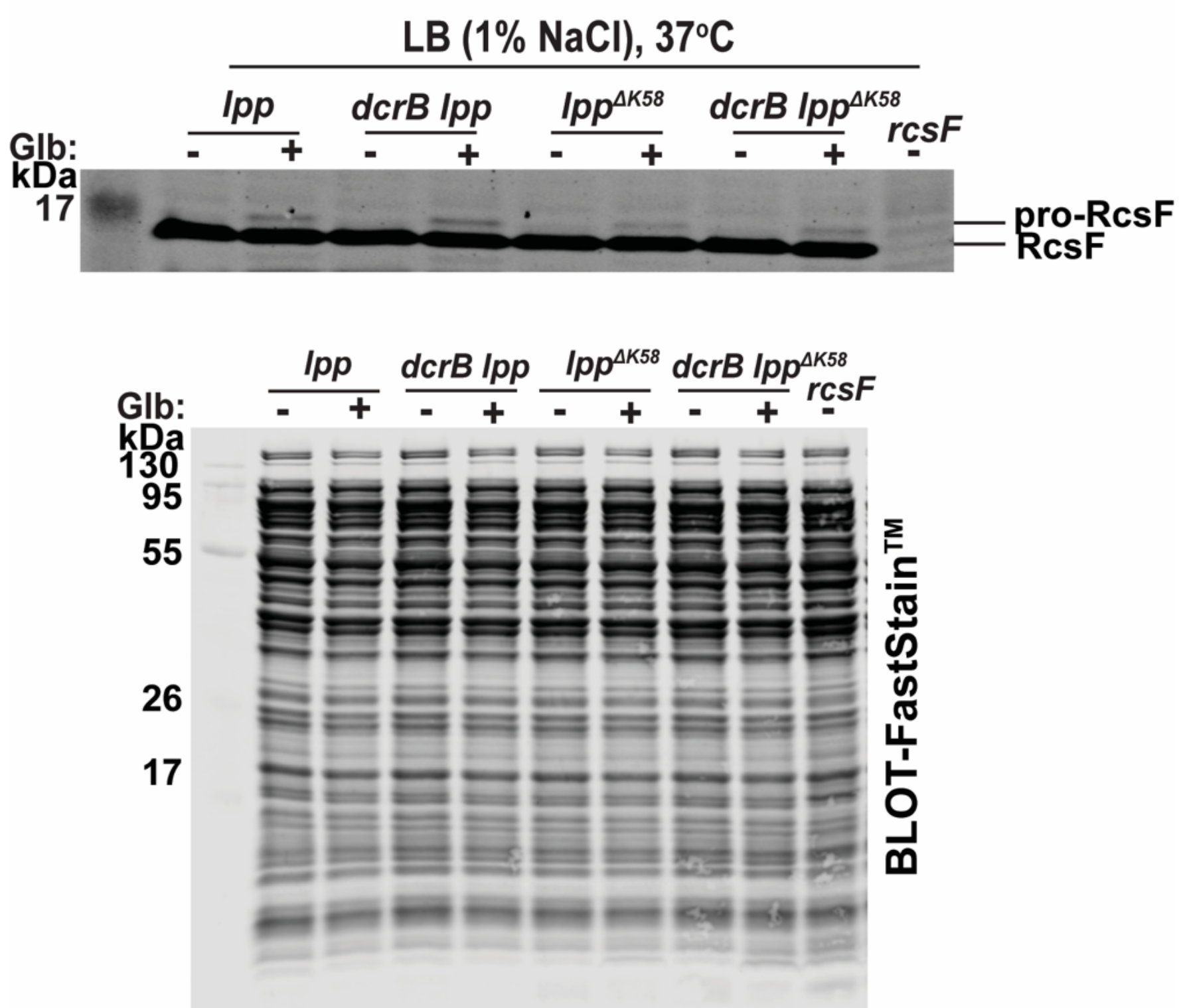


**Fig. S8. RcsF maturation in *dcrB* null cells.** Overnight cultures of WT and isogenic mutants *lpp* (EL603), *dcrB lpp* (EL656), *lpp*$^{\Delta K58}$ (AM1712) and *dcrB lpp*$^{\Delta K58}$ (AM1714) were subcultured to $OD_{600}$ = 0.03 in LB (1% NaCl) at 37°C in the presence and absence of 8 μg $mL^{-1}$ Glb (0.5X MIC of WT), then grown until mid-exponential phase. Cells were harvested, normalized to $OD_{600}$ value, and proteins separated by electrophoresis in 16% Tricine gels and immunoblotted for RcsF as described in the SI Methods. An *rcsF* null allele (EL1539) shows the specificity of the detected RcsF bands. Known and putative positions of RcsF and pro-RcsF are marked. Membrane staining for total protein using BLOT-FastStain$^{TM}$ (GBiosciences) is shown below and served as loading and transfer control.

**FIGURE S9**

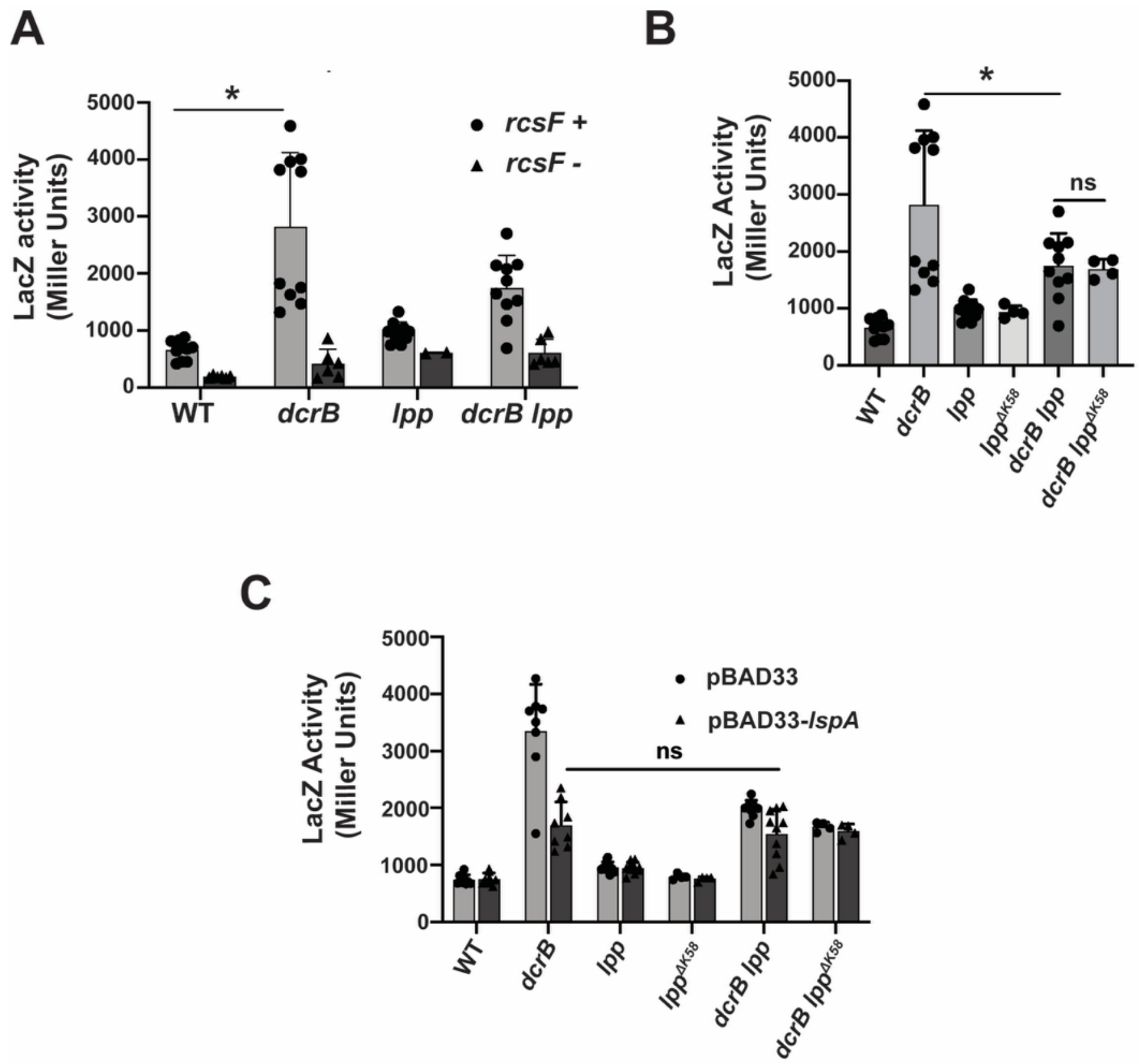


**Fig. S9. Rcs stress response is activated in *dcrB* null cells. (A)** A chromosomal *lacZ* fusion to a target gene promoter ($P_{rprA}$) of the Rcs stress response system was assayed for β-galactosidase activity in the TB28 (AM1249) and derivative mutant *dcrB*, *lpp*, and *dcrB lpp* backgrounds with *rcsF* (AM1260, MB145, MB111) or without (AM1817, MB113, MB147, MB115). Transcriptional activity was determined as described in the SI Methods. Mean and standard deviations are derived from triplicate readings of 3 or more independent cultures. Statistical analysis using an unpaired t-test between the respective strains with significant p-value <0.0001 marked as *. (**B)** Rcs activity was monitored as described in (A) in TB28 and derivative mutants *dcrB* (AM1260), *lpp* (MB145), *lpp*$^{\Delta K58}$ (MB281), *dcrB lpp* (MB111), and *dcrB lpp*$^{\Delta K58}$ (MB273). Mean and standard deviations are derived from triplicate readings of 4 or more independent cultures. Statistical analysis using an unpaired t-test between the respective strains with significant p-value = 0.035 and non-significant p-value = 0.8 marked as * or ns, respectively. **(C)**

Rcs activity was monitored as described in (A) in TB28 (AM1249) and derivative mutants *dcrB* (AM1260), *lpp* (MB145), *lpp*$^{\Delta K58}$ (MB281), *dcrB lpp* (MB111), and *dcrB lpp*$^{\Delta K58}$ (MB273) expressing either the empty pBAD33 vector or pBAD33-*lspA* (pMRB9). L-Ara was added at 0.02% at time of subculture to induce LspA expression. Statistical analysis using an unpaired t-test between the respective strains with a non-significant p-value = 0.8914 marked as ns.

## FIGURE S10

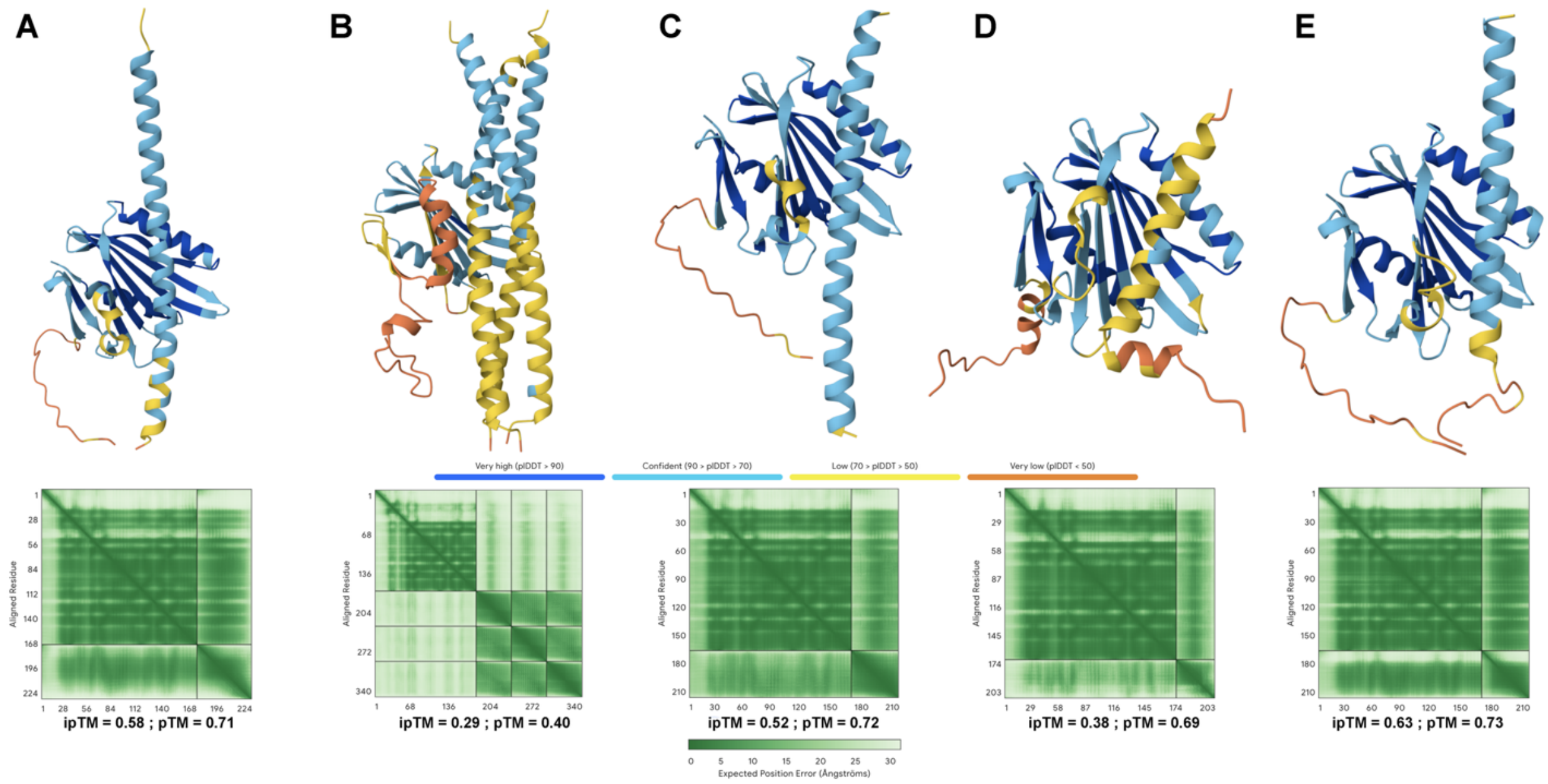


**Fig. S10. AF3 predictions of the interactions between DcrB and Lpp.** Predicted complexes between DcrB and Lpp monomer **(A)**, Lpp trimer **(B)**, Lpp$^{\Delta 49\text{-}58}$ monomer **(C)**, Lpp$^{\Delta 17\text{-}37}$ monomer **(D)**, and Lpp$^{L10P}$ monomer **(E)**, colored by the predicted local distance difference test (pLDDT). Predicted aligned error (PAE) plots are shown in green. The values of predicted template modelling (pTM) and interface predicted template modelling (ipTM) scores are also provided for each prediction.

**FIGURE S11**

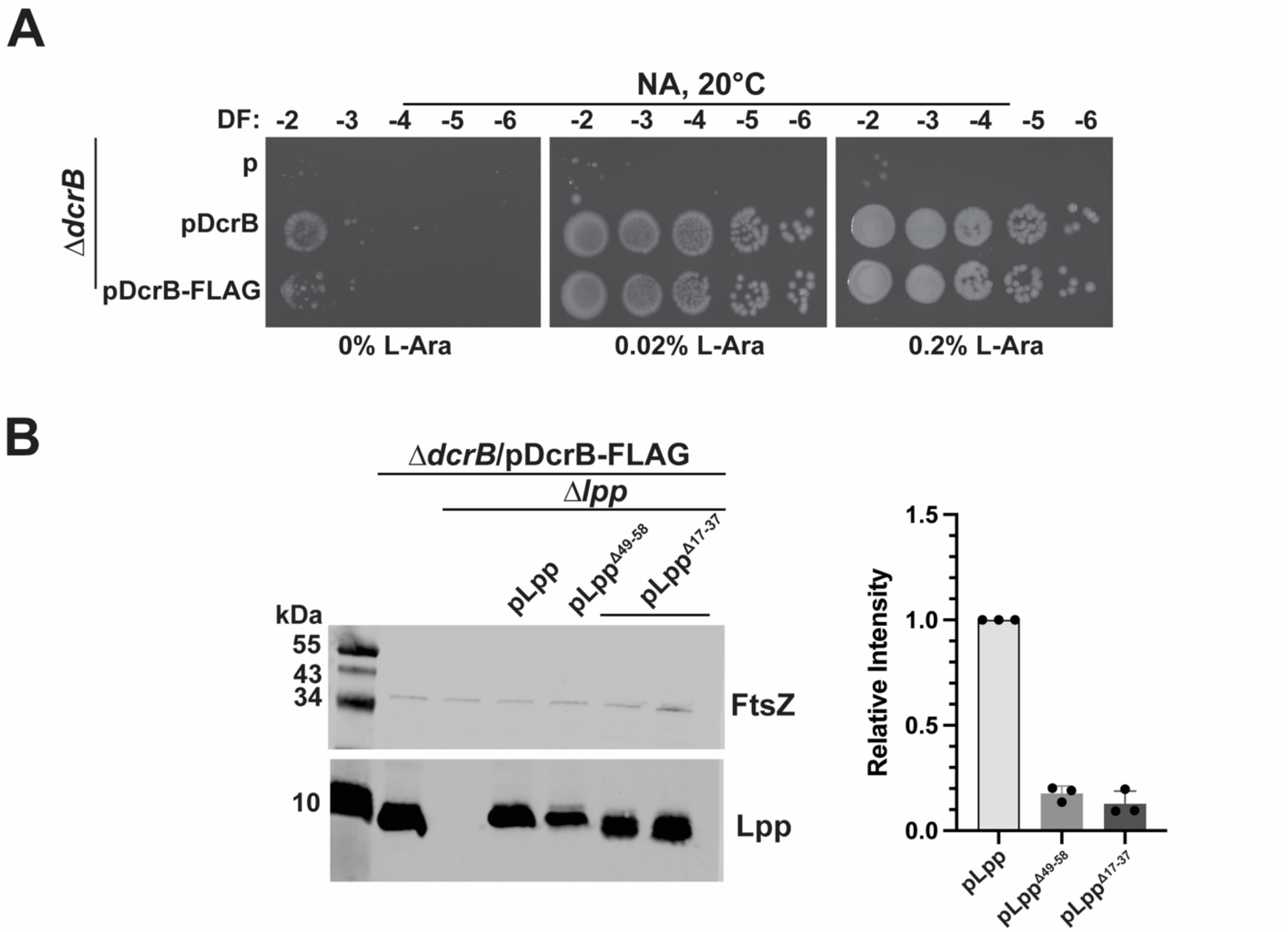


**Fig. S11. DcrB-FLAG functionality and expression of Lpp variants in crosslinking assays.** **(A)** DcrB-FLAG fusion is functional. Overnight cultures of a *dcrB* null (AM135) strain with the empty pBAD33 vector (p), or *dcrB* (pEL7), or *dcrB-FLAG* fusion (pMRB58) were grown in LB (1% NaCl) with Cm at 37°C, washed in NB, normalized to $OD_{600}$ in the same media, and serial dilutions were plated on NA plates supplemented with the labeled concentrations of the L-arabinose inducer. Plates were incubated for 72 h at 20°C before being imaged. Representative plate images are shown. **(B)** Immunoblots of pUC18-Lpp (pMRB61) or pUC18-Lpp variants (pMRB62, pMRB63) in the *dcrB lpp* double mutant background (EL656) expressing a *dcrB-FLAG* fusion (pMRB58) used in crosslinking assays reported in **Fig. 5B** of the main text. Proteins were grown, harvested by normalizing to $OD_{600}$, resolved in 16% Tris-glycine gel by electrophoresis, and probed with an anti-Lpp antibody as described in the Methods of the main text. FtsZ (~37 kDa)

served as a loading and transfer control. A representative image of three independent experiments is shown and the quantification of the relative intensities of the bands is reported on the right. For the *lpp*$^{\Delta 17\text{-}37}$ bearing strain, the same sample volume and 2X sample volume was loaded because of the reported poor detection by the Lpp antibody used in this study (1).

FIGURE S12

A

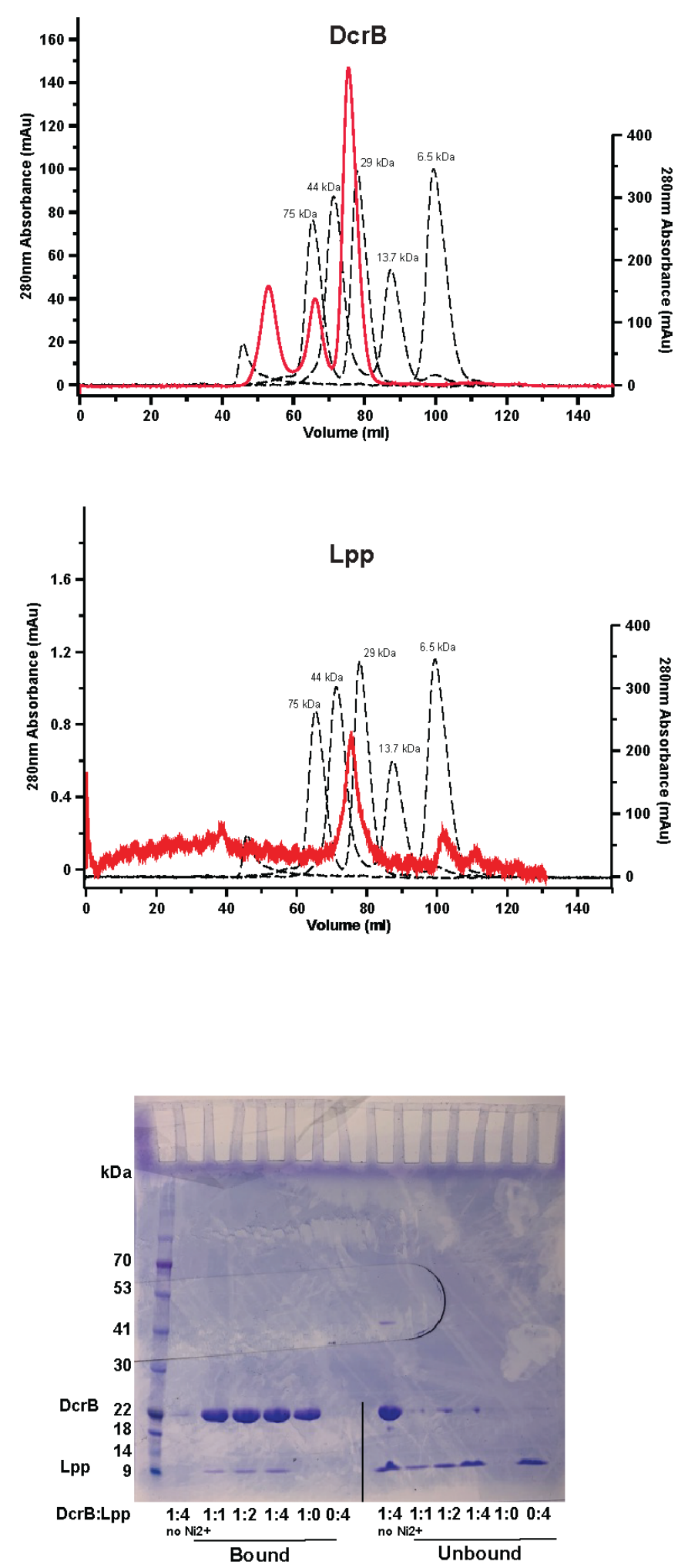


**Fig. S12. Purified DcrB and Lpp protein interactions (A)** Gel-filtration trace of purified 10xHis-FLAG-TEV-DcrB (red line), which runs through gel filtration (Superdex S75 Increase 10/300; GE Healthcare) predominantly as a monomer (right peak) with a small portion oligomerizing (middle peak) is shown. Below is the gel filtration trace of purified tagless Lpp (red line), which runs through gel filtration (Superdex S75 Increase 10/300) as an oligomer. **(B)** The uncropped SDS-PAGE gel stained with Coomassie blue shown in **Fig. 5C** of the main text.

## MATERIALS AND METHODS

### Plasmid construction

**pEL01 (pBAD33-*lspA*-3xFLAG)** was constructed by amplifying off of the *lspA-3xFLAG* chromosomal integrant DNA with primers MB33F and EL180R and cloning into the KpnI/HindIII sites of pBAD33.

**pEL4 (pDSW206-*dcrB*$_{\mathbf{OM}}$)** was constructed by amplifying the entire pEL3 (pDSW206-*dcrB*) plasmid with primers EL36F and EL37R with the desired D21S mutation. The correct clone was mutagenized with EL63F and EL64R primers to obtain the D22S variant. The double mutant clones (D21S, D22S) were verified by sequencing, and re-ligated into the same SacI/SalI sites of pDSW206 vector.

**pEL5 (pDSW206-*dcrB-mScar*)** *dcrB* was first cloned into pHCL147 without the stop codon using EL89F and EL43R primers and NdeI/BamHI sites. Next, AM3F and EL196R primers were used to amplify the sequenced *dcrB-mScar* fusion from pHCL147 and cloned into the SacI/SalI sites of pDSW206.

**pEL6 (pDSW206-*dcrB*$_{OM}$*-mScar*)** was constructed by amplifying the *dcrB*$_{OM}$ fragment from pEL4 (pDSW206-*dcrB*$_{OM}$) plasmid template with EL197F and EL43R primers. A SacI/BamHI digest of the pEL5 (pDSW206-*dcrB-mScar*) backbone was gel purified and ligated with the newly synthesized *dcrB*$_{OM}$ fragment with matching SacI/BamHI overhangs.

**pEL13 and pEL14 (pDSW204-*oppB*$_{TM}$*-lpp* and pDSW204-*oppB*$_{TM}$*-lpp*$^{\Delta K58}$)** were constructed via iVEC cloning (2). First, pDSW204 plasmid was digested with SacI/SalI. Then DNA sequence corresponding to amino acid residues 1-30 of the OppB N-terminus and transmembrane (TM) domain were amplified from the MG1655 genome using EL177F and EL178R primers with

overhangs for both the pDSW204 SacI site and DNA sequence corresponding to Lpp amino acid residues 22-78 of the full-length protein (double underlined in the primer sequence). DNA corresponding to Lpp residues 22-78 or 22-77 (ΔK58) were amplified with similar overhangs for either the $OppB_{TM}$ domain (double underlined in the primer sequence) or the pDSW204 SalI cut site using the following primers: EL179F and EL126R primers (with the K58 codon) or EL127R (without the K58 codon). The amplified PCR fragments ($OppB_{TM}$, Lpp22-78, and Lpp22-77) were assembled into the SacI/SalI digested pDSW204 backbone via iVEC.

**pEL15 and pEL16 (pDSW204-*$malF_{TM}$-lpp* and pDSW204-*$malF_{TM}$-$lpp^{\Delta K58}$*)** were cloned similarly to $OppB_{TM}$-Lpp via amplification of DNA sequence corresponding to both $MalF_{TM}$ amino acid residues 1-36 and Lpp residues 22-78 and 22-77 with overhangs for iVEC cloning. DNA coding for MalF residues 1-36 was amplified from the MG1655 genome using the following primers: EL123F primer to amplify the sequence coding for MalF residues 1-36 with overhangs for pDSW204 SacI site and EL204R primer to amplify DNA coding for MalF residue 36 with overhangs for Lpp (double underlined in the primer sequence). To amplify Lpp with overhangs for MalF DNA corresponding to amino acid residues 1-36 (double underlined in the primer sequence), EL205F primer was used with the aforementioned EL126R or EL127R primers for amplification of Lpp with or without K58 codon with overhangs for the pDSW204 backbone. After amplification of DNA fragments corresponding to MalF residues 1-36 and Lpp residues 22-77 and 22-78, these fragments were cloned via iVec into SacI/SalI-digested pDSW204 vector.

**pEL17, pEL42, and pEL19 (pDSW204-*lpp-mScar*, pDSW204-*$lpp^{C21G}$-mScar,* and pDSW204-*$oppB_{TM}$-lpp-mScar*)** were constructed as follows. Primers EL173F and EL174R were used to amplify previously mutagenized *$lpp^{C21G}$* sequence without its stop codon from pDSW204-*$lpp^{C21G}$* plasmid and cloned into the NdeI/BamHI restriction sites of pHCL147 to create a C-terminal fusion

to mScarlet-I. Next, the *lpp*$^{C21G}$-*mScar* DNA was amplified off of pHCL147-*lpp*$^{C21G}$-*mScar* with primer pair EL90F and EL196R and ligated into similarly digested SacI/SalI pDSW204 vector to construct pEL42. To construct pEL17, primer pair EL90F and EL174R were used to amplify *lpp* from MG1655 genomic DNA without a stop codon. The pDSW204-*lpp*$^{C21G}$-*mScar* backbone was digested with SacI/BamHI enzymes, and the pDSW204-*lpp*-*mScar* fusion was constructed. To construct pEL19, primer EL197F was used in conjunction with primer EL174R to amplify $oppB_{TM}$-*lpp* without its stop codon. The pDSW204-*lpp*$^{C21G}$-*mScar* backbone was digested with SacI/BamHI enzymes, and the pDSW204-$oppB_{TM}$-*lpp*-*mScar* fusion was created.

**pEL39 and pEL40 (pDSW204-*lpp*$^{L10P, \Delta K58}$ and pUC18-*lpp*$^{L10P}$*)*** were constructed from previously engineered plasmids pDSW204-*lpp*$^{\Delta K58}$ (pEL37) and pDSW204-*lpp* (pEL36) by amplifying the entire plasmid using the following mutagenesis primers: EL211F and EL212R. The PCR products were then transformed into iVEC cells for cloning, verified by sequencing, digested with SacI/SalI and ligated into fresh pDSW204 backbone vectors (or, in the case of pEL40, into pUC18).

**pFR2 and pFR3 (pDSW204-*lpp*$^{C21G}$ and pDSW204-*lpp*$^{C21G, \Delta K58}$)** were constructed from previously engineered plasmids pDSW204-*lpp* (pEL36) and pDSW204-*lpp*$^{\Delta K58}$ (pEL37). Next, site directed mutagenesis primer pair EL134F and EL135R was used to PCR pDSW204-*lpp* and pDSW204-*lpp*$^{\Delta K58}$ plasmids to generate pDSW204-*lpp*$^{C21G}$ *or lpp*$^{C21G, \Delta K58}$ variants. The *lpp*$^{C21G}$ and *lpp*$^{C21G, \Delta K58}$ mutant inserts were digested with SacI/SalI enzymes and ligated into fresh pDSW204 vector.

**pMB1 (pNYCOMPS-Nterm-*dcrB*)** was constructed by amplifying DNA corresponding to amino acid residues 21-185 of full length DcrB using primers MRB1F and MRB2R with the necessary

overhangs for Ligation Independent Cloning (LIC) as previously described (3). The amplified product was then inserted into the vector pNYCOMPS-N-term using LIC.

**pMB2 (pMCSG9-*lpp*)** was constructed by amplifying DNA corresponding to amino acid residues 22-78 of Lpp using primers MRB3F and MRB4R. The amplified product was inserted into the vector pMCGS9 using LIC.

**pMRB58 (pBAD33-*dcrB-3xFLAG*)** was constructed by amplifying *dcrB*-FLAG off of MG1655 *dcrB*-3xFLAG genomic DNA with primers AM3F and DB21R with SacI/SalI overhangs and cloning into the same sites in pBAD33.

**pMRB63 and pEL41 (pUC18-*lpp*$^{\Delta 17\text{-}37}$ and pDSW204-*lpp*$^{\Delta 17\text{-}37,\ \Delta K58}$)** were constructed by using primer pair EL90F and EL91R (pMRB63) or EL96R (pEL41) to amplify *lpp* DNA corresponding to the deletion of amino acid residues 17-37 of the mature protein off of the pIDTSMART-AMP-*lpp*$^{\Delta 17\text{-}37}$ (Integrated DNA Technologies) plasmid template with SacI/SalI overhangs and cloned into the same sites in pUC18 or pDSW204.

**Chromosomal integrant construction**

***dcrB-3xFLAG* chromosomal integrant** was created via PCR amplification of the Kan$^R$ region of the pSUB11 plasmid (4) with primer pair EL128F and EL128R containing 5' overhangs for the end of *dcrB* to replace the stop codon with the 3x-FLAG tag by Red recombination (4, 5).

***lspA-3xFLAG* chromosomal integrant** was created via PCR amplification of the Kan$^R$ region of the pSUB11 plasmid (4) with primers MB44F and MB45R containing 5' overhangs (double underlined in the primer sequence) for the end of *lspA* to replace the stop codon with the 3x-FLAG tag by Red recombination (4, 5).

**Note:** Plasmids whose construction details are not described above were generated using routine techniques with MG1655 genomic DNA as template. They are included in **Table S4** listing all the primers used in the study.

**iVEC cloning**

iVEC cloning was conducted as described (2). Briefly, 1.5 ml overnight cultures of appropriate strains grown at 37°C for 16-20 h were chilled on ice for 10 min and pelleted at 4°C. Separately, 50 ng of digested vector was mixed with 100 ng of PCR inserts in 100 µL of ice-cold Transformation and Stock solution (TSS: L broth, DMSO, PEG8000, $MgSO_4$, and 80% glycerol). Cell pellets were resuspended in TSS solution, immediately submerged in liquid nitrogen, and allowed to thaw on ice for 10 min, at which point the tubes were vortexed briefly and incubated on ice for an additional 10 min. An aliquot of 1 mL LB (0.5% NaCl) was added to the mix and placed in a 37°C water-bath for 45 min. Transformed cells were pelleted and plated on selective plates.

**LIC cloning**

For LIC (3), PCR reactions were done using the KOD Hot Start DNA Polymerase (EMD Millipore) using the manufacturer's protocol. Amplified products were cleaned using a PCR cleanup kit (Qiagen) before being prepared for insertion by adding 4 µL of each product to a mix of NE buffer (2 µL), 2.5 mM dCTP, T4 DNA polymerase, and $H_2O$ to a volume of 20 µL and incubating the mixture overnight at 20°C. The following day, 2 µL of vector and 4 µL of insert were combined and incubated at 22°C for 1 h. Two µL of 25 mM EDTA was then added and the mixture incubated for an additional 5 min at 22 °C. Plasmids were then transformed into DH10BT1$^{R}$ competent cells (NEB) using heat shock.

**Lgt depletion**

Lgt depletion strains were constructed as described previously (6) in the lab stock of MG1655. Strains bearing the pCHAP9231 *lgt* shelter plasmid were grown as described (7) overnight in Lgt-replete conditions in LB (1% NaCl) supplemented with Cm and 0.2% L-Ara at 37°C. After 16-18 h, cells were pelleted, washed 3x in LB (1% NaCl) before sub-culturing to an $OD_{600}$ ~0.05 in LB

(1% NaCl) with Cm and 0.2% glucose or 0.005% or 0.2% L-arabinose for growth in Lgt-deplete and -replete conditions respectively, in a 96-well microtiter plate. Plates were incubated and growth monitored via $OD_{600}$ recorded in a Spark® (Tecan) plate reader over the course of 16 h at 37°C.

**β-galactosidase assays**

Assays were performed essentially as described (8). Strains grown overnight under permissive conditions were subcultured 1:150 in LB (0% NaCl) and grown at 20°C for 11-13 h or in LB (1% NaCl) at 37°C for 2 h. A 200 µL aliquot of each culture was transferred to a 96-well flat bottom plate and $OD_{595}$ values were read using a SpectraMAX M2 (Molecular Devices) plate reader. Next, 100 µl of cultures were transferred to a new 96-well plate and mixed with 10 µl of PopCulture reagent (EMD Millipore) containing 0.4 units $\mu l^{-1}$ of rLysozyme (Novagen) and incubated at room temperature for 30 min to lyse the cells. Thirty µl of lysate was transferred to a new 96-well plate, mixed with 150 µl of Z-buffer (8.53 g of $Na_2HPO_4$, 5.5 g $NaH_2PO_4$, 0.75 g of KCl, and 0.246 g $MgSO_4$ in 1L) containing O-nitrophenyl-β-galactoside (ONPG, 2 mg $ml^{-1}$) and 0.2% β-mercaptoethanol (BME), and placed in the plate reader maintained at 28°C. The $OD_{415}$ values were read every minute for an hour. Miller units were derived using a modified equation as previously described (8).

**Membrane fractionation**

Membrane fractionation was performed as previously described (9) with minor modifications. Overnight cultures in standard laboratory conditions were subcultured 1:100 in 250 mL of NB at 20°C until mid-log ($OD_{600}$ ~0.5 - 0.7). Cells were washed once with 10 mM Tris-HCl (pH 8.0) before being resuspended in 20 mL of cold 10 mM Tris-HCl (pH 8.0) containing 20% sucrose (wt/wt), Benzonase (EMD Millipore), and Pierce protease inhibitor (ThermoFisher). Cells were lysed by

passing once through a French Pressure Cell Press (ThermoFisher) at 8,000 psi. Lysates were centrifuged at 3,000 x*g* for 15 min at 4 °C to remove cell debris, and EDTA was added to the cleared lysate to a 1 mM final concentration. In an Ultra-Clear open top tube (Beckman Coulter), 4 mL of lysate was layered on top of a two-step sucrose gradient of 5 mL of 40% sucrose on top of 1.5 mL of 65% sucrose. Sucrose solutions were dissolved in 10 mM Tris-HCl, pH 8.0 (wt/wt). Samples were centrifuged at 35,000 rpm for 18 h at 4°C in a Beckman SW 40 Ti rotor in an Optima XE-100 Ultracentrifuge (Beckman Coulter). Fractions were collected manually in 1 mL increments from the top using wide-bore pipette tips and used for further analysis.

**Immunoblot analyses**

Immunoblotting was conducted as described in the Methods of the main text with minor modifications. For LspA-FLAG detection, protein samples were resuspended in 1X SDS-sample buffer containing 4% BME and resolved by electrophoresis in 12.5% SDS-PAGE gels. Normalization of LspA levels was done by comparison to the loading control FtsZ. FtsZ was probed via a primary rabbit anti-FtsZ polyclonal antibody (GenScript) at 1:10,000 at 4°C overnight. Detection for RcsF and LamB was done essentially as described for Lpp in the main text. An anti-RcsF rabbit polyclonal antibody was used at 1:10,000 dilution for 2.5 h at room temperature and an anti-LamB rabbit polyclonal primary at 1:30,000 at 4°C overnight was used for detection.

**TABLE S1. Predicted cleavage of Lpp variants used in the study.**[a]

| Variants | Cleavage by SPI (%) | Cleavage by SPII (%) | Cleavage by pilin-like signal peptide (Sec/SPIII) (%) |
|---|---|---|---|
| WT Lpp | 0 | 100 | 0 |
| $Lpp^{C21G}$ | 49.8 | 50.1 | 0.01 |
| $OppB_{TM}$-Lpp | 0.06 | 0.04 | 0.72 |
| $MalF_{TM}$-Lpp | 0.04 | 0.04 | 13.63 |

[a] Obtained from SignalP-6.0 site (https://services.healthtech.dtu.dk/services/SignalP-6.0).

**Table S2. Strains and plasmids reported in the main text.**

| Strain/Plasmid | Genotype/Description | Source/Reference[a] |
|---|---|---|
| MG1655 | K12 F⁻ λ⁻ *ilvG⁻ rfb-50 rph-1* | Laboratory collection |
| TB28 | MG1655 *lacIZYA::frt* | (10) |
| BL21pLysS | F⁻ *ompT gal dcm lon hsdSB rB⁻ mB⁻* λDE3 *lacI lacUV5-T7 gene 1 ind1 sam7 nin5* | Novagen |
| T7 Express | *fhuA2 lacZ::T7 gene1 [lon] ompT gal sulA11 R(mcr-73::miniTn10*--Tet$^S$*)2 [dcm] R(zgb-210::Tn10*-Tet$^S$*) endA1 Δ(mcrC-mrr)114::IS10* | NEB |
| AM135 | MG1655 *dcrB::frt* | (8) |
| AM814 | MG1655 *dcrB::frt lpp::kan*$^R$ | (11) |
| AM949 | MG1655 *lpp::kan*$^R$ | (11) |
| AM1712 | MG1655 *lpp*$^{\Delta K58}$ *ynhG(ldtE)::kan*$^R$ | (11) |
| AM1714 | AM135 *lpp*$^{\Delta K58}$ *ynhG*(*ldtE)::kan*$^R$ | (11) |
| EL603 | MG1655 *lpp::frt* | |
| EL656 | MG1655 *dcrB::frt lpp::frt* | |
| pBAD33 | p15A, Cm$^R$, *araC* $P_{ara}$::empty | (12) |
| pDSW204 | pTrc99a, Amp$^R$, promoter attenuated (-35, TTGACA to TTTACA | (13) |
| pDSW206 | pTrc99a, Amp$^R$, promoter attenuated (-10, TATAAT to CATTAT); (-35, TTGACA to TTTACA) | (13) |
| pHCL147 | Tet$^R$, *lacI*-$P_{lac}$-*dsbA*$^{SS}$*-mScar* | (14) |
| pNYCOMPS-Nterm | pET24, Kan$^R$,10xHis-FLAG-TEV | (15) |

| | | |
|---|---|---|
| pMCSG9 | pBR322, $Amp^R$, 6xHis-MBP-TEV | (16) |
| pSUB11 | pUC18, $Kan^R$ $Amp^R$, *3xFLAG* cloned adjacent to pKD4-derived FRT-Kan-FRT | (4) |
| pTrc99a | pBR/ColE1, $Amp^R$, *lacI* $P_{lac}$::empty | (17) |
| pUC18 | pMB1/ColE1, AmpR, *lacZ*α | Stratagene |
| pAM8 | pDSW206-*lgt* | (11) |
| pEL3 | pDSW206-*dcrB* | |
| pEL4 | pDSW206-$dcrB_{OM}$ | |
| pEL5 | pDSW206-*dcrB-mScar* | |
| pEL6 | pDSW206-$dcrB_{OM}$-*mScar* | |
| pEL8 | pDSW206-*lspA* | |
| pEL9 | pDSW206-*lnt* | |
| pEL10 | pDSW206-*lolCDE* | |
| pEL13 | pDSW204-$oppB_{TM}$-*lpp* | |
| pEL14 | pDSW204-$oppB_{TM}$-$lpp^{\Delta K58}$ | |
| pEL15 | pDSW204-$malF_{TM}$-*lpp* | |
| pEL16 | pDSW204-$malF_{TM}$-$lpp^{\Delta K58}$ | |
| pEL17 | pDSW204-*lpp-mScar* | |
| pEL19 | pDSW204-$oppB_{TM}$-*lpp-mScar* | |
| pEL37 | pDSW204-$lpp^{\Delta K58}$ | |
| pEL39 | pDSW204-$lpp^{L10P,\ \Delta K58}$ | |
| pEL41 | pDSW204-$lpp^{\Delta 17-37,\ \Delta K58}$ | |
| pEL42 | pDSW204-$lpp^{C21G}$-*mScar* | |
| pFR1 | pTrc99a-*ldtF* | |
| pFR2 | pDSW204-$lpp^{C21G}$ | |

| | |
|---|---|
| pFR3 | pDSW204-*lpp*$^{C21G,\ \Delta K58}$ |
| pMB1 | pNYCOMPS-N-term-*dcrB* |
| pMB2 | pMCSG9-*lpp* |
| pMRB58 | pBAD33-*dcrB-3xFLAG* |
| pMRB61 | pUC18-*lpp* |
| pMRB62 | pUC18-*lpp*$^{\Delta 49\text{-}58}$ |
| pMRB63 | pUC18-*lpp*$^{\Delta 17\text{-}37}$ |

[a] This study unless otherwise indicated.

**Table S3. Additional strains and plasmids reported in the supporting information.**

| Strain/Plasmid | Genotype/Description | Source/Reference[a] |
|---|---|---|
| AM1249 | TB28 *nadA*::Tn10 attλ::$P_{rprA\text{-}lacZ}$ | (8) |
| AM1817 | AM1249 *rcsF::kan*$^{R}$ | (8) |
| AM1260 | TB28 *dcrB::frt nadA*::Tn10 attλ::$P_{rprA\text{-}lacZ}$ | (8) |
| EL627 | MG1655 pCHAP9231 | |
| EL632 | MG1655 *lgt::kan*$^{R}$ pCHAP9231 | |
| EL634 | AM135 *lgt::kan*$^{R}$ pCHAP9231 | |
| EL668 | EL603 *lgt::kan*$^{R}$ pCHAP9231 | |
| EL670 | EL656 *lgt::kan*$^{R}$ pCHAP9231 | |
| EL1231 | MG1655 pEL01 | |
| EL1242 | MG1655 *lspA::kan*$^{R}$ pEL01 | |
| EL1244 | AM135 *lspA::kan*$^{R}$ pEL01 | |
| EL1247 | EL603 *lspA::kan*$^{R}$ pEL01 | |
| EL1248 | EL656 *lspA::kan*$^{R}$ pEL01 | |
| EL1539 | MG1655 *rcsF::kan*$^{R}$ | |
| MB111 | AM1260 *lpp::frt* | |
| MB113 | AM1260 *rcsF::kan*$^{R}$ | |
| MB115 | MB111 *rcsF::kan*$^{R}$ | |
| MB145 | AM1249 *lpp::frt* | |
| MB147 | MB145 *rcsF::kan*$^{R}$ | |
| MB270 | MG1655 *lspA*::*lspA-3xFLAG-kan*$^{R}$ | |
| MB271 | AM135 *lspA*::*lspA-3xFLAG-kan*$^{R}$ | |
| MB273 | AM1260 *lpp*$^{\Delta K58}$ *ldtE::kan*$^{R}$ | |
| MB281 | AM1249 *lpp*$^{\Delta K58}$ *ldtE::kan*$^{R}$ | |

| | | |
|---|---|---|
| pCHAP9231 | pBAD18s-Cm-*lgt*$^{WT}$-c-myc$_2$ | (6) |
| pEL01 | pBAD33-*lspA-3xFLAG* | |
| pEL7 | pBAD33-*dcrB* | |
| pEL36 | pDSW204-*lpp* | |
| pEL40 | pUC18-*lpp*$^{L10P}$ | |
| pMRB9 | pBAD33-*lspA* | |

[a] This study unless otherwise indicated.

**Table S4. Primers used in the study.**

| Plasmid Name/Description | Primer Name | Primers (5' to 3')* | RE Sites^ |
|---|---|---|---|
| pEL01 (pBAD33-*lspA-3xFLAG*) | MB33F | CGCGCGGTACCAGGAGGAAGGCG**ATG**AGTCAATCGATCTGTTCAAC | KpnI |
| | EL180R | CGCGCAAGCTTCTATTTATCGTCGTCATCTTTGTAGTC | HindIII |
| pEL3 (pDSW206-*dcrB*) | AM3F | CGCGCGAGCTCAGGAGGAAGGCG**ATG**CGCAATCTGGTTAAATATG | SacI |
| | AM4R | CGCGCGTCGACTTACTGAATAACCAGCGTATTAATGATG | SalI |
| pEL4 (pDSW206-*dcrB*$_{OM}$) | AM3F | " | SacI |
| | AM4R | " | SalI |
| | EL36F | GGGCTTGCGGCCTGT**TCT**GATAAAGACACTAACG | N/A |
| | EL37R | CAGGCCGCAAGCCCCATAAC | " |
| | EL63F | GGCTTGCGGCCTGTTCT**AGC**AAAGACACTAACGCTACG | " |
| | EL64R | GAACAGGCCGCAAGCCCC | " |
| pEL5 (pDSW206-*dcrB-mScar*) | EL89F | CGCGCCAT**ATG**CGCAATCTGGTTAAATATGTCGG | NdeI |
| | EL43R | CGCGCGGATCCCTGAATAACCAGCGTATTAATGATGTTTTCTGCG | BamHI |
| | AM3F | listed above | SacI |
| | EL196R | CGCGCGTCGACTTACTTATACAGTTCATCCATACCTC | SalI |
| pEL6 (pDSW206-*dcrB*$_{OM}$*-mScar*) | EL197F | CGCGCGAGCTCAGGAGGAAGG | SacI |
| | EL43R | listed above | BamHI |
| pEL7 (pBAD33-*dcrB*) | AM3F | " | SacI |
| | AM4R | " | SalI |
| pEL8 (pDSW206-*lspA*) | EL115F | CGCGCGAATTCAGGAGGAAGGCG**ATG**AGTCAATCGATCTGTTCAAC | EcoRI |
| | EL116R | CGCGCGTCGACTTATTGTTTTTTCGCTCTAGAAGGC | SalI |
| pEL9 (pDSW206-*lnt*) | EL136F | CGCGCGAGCTCAGGAGGAAGGCG**ATG**GCTTTTGCCTCATTAATTG | SacI |
| | EL137R | CGCGCTCTAGATTATTTACGTCGCTGACGC | XbaI |
| pEL10 (pDSW206-*lolCDE)* | EL183F | CGCGCGAATTCAGGAGGAAGGCG**ATG**TACCAACCTGTCGCTC | EcoRI |
| | EL184R | CGCGCTCTAGATTACTGGCCGCTAAGGACTC | XbaI |
| pEL13 & 14 (pDSW204-*oppB*$_{TM}$-*lpp* $^{+/- K58}$) | EL177F | CCATGGAATTCGAGCTCAGGAGGAAGGCG**ATG**TTAAAATTTATTCTACGTCGCTGTCTGG | SacI |
| | EL178R | ATCGATTTTAGCGTTGCTGGACGCGAGGCGCATCATAAAG | N/A |

| | | | |
|---|---|---|---|
| | EL179F | GTTCTTTATGATGCGCCTCGCGTCCAGCAACGCTAAAATCGATC | " |
| | EL126R | CAGCCAAGCTTGCATGCCTGCAGGTCGACTTACTTGCGGTATTTAGTAGCCATG | SalI |
| | EL127R | GCCAAGCTTGCATGCCTGCAGGTCGACTTAGCGGTATTTAGTAGCCATGTTGTC | " |
| pEL15 & 16 (pDSW204-*malF*$_{TM}$-*lpp* $^{+/- K58}$) | EL123F | CCATGGAATTCGAGCTCAGGAGGAAGGCG**ATG**GATGTCATTAAAAAGAAACATTG | SacI |
| | EL204R | ATCGATTTTAGCGTTGCTGGATGCGTACATTAAAACAACAAGG | N/A |
| | EL205F | GTTACCTTGTTGTTTTAATGTACGCATCCAGCAACGCTAAAATCGATC | " |
| | EL126R | listed above | SalI |
| | EL127R | " | " |
| pEL17 (pDSW204-*lpp-mScar*) | EL90F | CGCGCGAGCTCAGGAGGAAGGCG**ATG**AAAGCTACTAAACTGGTACTG | SacI |
| | EL174R | CGCGCGGATCCCTTGCGGTATTTAGTAGCCATG | BamHI |
| pEL19 (pDSW204-*oppB*$_{TM}$-*lpp-mScar)* | EL197F | listed above | SacI |
| | EL174R | " | BamHI |
| pEL36 (pDSW204-*lpp*) | EL90F | " | SacI |
| | EL91R | CGCGCGTCGACTTACTTGCGGTATTTAGTAGCC | SalI |
| pEL37 (pDSW204-*lpp*$^{\Delta K58}$) | EL90F | listed above | SacI |
| | EL96R | CGCGCGTCGACTTAGCGGTATTTAGTAGCCATGTTG | SalI |
| pEL39 (pDSW204-*lpp*$^{L10P,\ \Delta K58}$) | EL90F | listed above | SacI |
| | EL96R | " | SalI |
| | EL211F | CGCTAAAATCGATCAGC**C**GTCTTCTGACGTTCAGACTC | N/A |
| | EL212R | CTGATCGATTTTAGCGTTGCTGGAGC | " |
| pEL40 (pUC18-*lpp*$^{L10P}$) | EL90F | listed above | SacI |
| | EL91R | " | SalI |
| | EL211F | " | N/A |
| | EL212R | " | " |
| pEL41 (pDSW204-*lpp*$^{\Delta 17–37,\ \Delta K58}$) | EL90F | " | SacI |
| | EL96R | " | SalI |
| pEL42 (pDSW204-*lpp*$^{C21G}$-*mScar*) | EL173F | CGCGCCAT**ATG**AAAGCTACTAAACTGGTACTG | NdeI |
| | EL174R | listed above | BamHI |
| | EL90F | " | SacI |
| | EL196R | " | SalI |
| pFR1 (pTrc99a-*ldtF*) | EL201F | CGCGCGAGCTCAGGAGGAAGGCG**ATG**CGTAAAATCGCATTAATTC | SacI |
| | EL202R | CGCGCGTCGACTTATTTTGCCTCGGGGAG | SalI |

| | | | |
|---|---|---|---|
| pFR2 & 3 (pDSW204-*lpp*$^{C21G\ +/-\ K58}$) | EL90F | listed above | SacI |
| | EL91R | " | SalI |
| | EL96R | " | SalI |
| | EL134F | CTCTGCTGGCAGGT**G**GCTCCAGCAACGCTA | N/A |
| | EL135R | ACCTGCCAGCAGAGTAGAACCCAGG | " |
| pMB1 (pNYCOMPS-N-term-*dcrB*) | MRB1F | TATTTTCAATCCTACGTAGATGATAAAGACA CTAACGCTAC | " |
| | MRB2R | CCCTCAATATTATACGGGTTACTGAATAACC AGCGTATTAATG | " |
| pMB2 (pMCSG9-*lpp*) | MRB3F | TACTTCCAATCCAATGCCTGCTCCAGCAACG CTAAAATCG | " |
| | MRB4R | TTATCCACTTCCAATGTTACTTGCGGTATTTA GTAGCC | " |
| pMRB9 (pBAD33-*lspA*) | MB33F | listed above | " |
| | MB34R | CGCGCAAGCTTTTATTGTTTTTCGCTCTAG AAGGC | HindIII |
| pMRB58 (pBAD33-*dcrB-3xFLAG*) | AM3F | listed above | SacI |
| | DB21R | CGCGCGTCGACCTATTTATCGTCGTCATCTT TGTAGTC | SalI |
| pMRB61 (pUC18-lpp) | EL90F | listed above | SacI |
| | EL91R | " | SalI |
| pMRB62 (pUC18-lpp$^{\Delta 49\text{-}58}$) | EL90F | " | SacI |
| | MB80R | CGCGCGTCGACTTAACGCTGGTTAGCACGA GC | SalI |
| pMRB63 (pUC18-lpp$^{\Delta 17\text{-}37}$) | EL90F | listed above | SacI |
| | EL91R | " | SalI |
| *dcrB-3xFLAG* PCR fragment | EL128F | CAGAAAACATCATTAATACGCTGGTTATTCA GGACTACAAAGACCATGACGG | N/A |
| | EL128R | CACGTCCCTGAGGCCGCCTCATCATCTTAAA ACCATATGAATATCCTCCTTAGTTCC | " |
| *lspA-3xFLAG* PCR fragment | MB44F | GAAGGTTTTTTGCCTTCTAGAGCGAAAAAAC AAGACTACAAAGACCATGACGG | N/A |
| | MB45R | CCGGATAAGATGCGTCAGCATCGCATCCGG CAGGGTTTACATATGAATATCCTCCTTAGTT CC | " |

* Restriction sites are underlined, translational start is in bold, and mutagenesis sites are both underlined and in bold.

^RE = Restriction Enzyme, N/A = Not applicable.

## SI REFERENCES


1. Cowles CE, Li Y, Semmelhack MF, Cristea IM, Silhavy TJ. 2011. The free and bound forms of Lpp occupy distinct subcellular locations in *Escherichia coli*. Mol Microbiol 79:1168-1181.
2. Nozaki S, Niki H. 2019. Exonuclease III (XthA) enforces *in vivo* DNA cloning of *Escherichia coli* to create cohesive ends. J Bacteriol 201:e00660-18.
3. Bruni R, Kloss B. 2013. High-throughput cloning and expression of integral membrane proteins in *Escherichia coli*. Curr Protoc Protein Sci 74:29.6.1-29.6.34.
4. Uzzau S, Figueroa-Bossi N, Rubino S, Bossi L. 2001. Epitope tagging of chromosomal genes in *Salmonella*. Proc Natl Acad Sci U S A 98:15264-9.
5. Datsenko KA, Wanner BL. 2000. One-step inactivation of chromosomal genes in *Escherichia coli* K-12 using PCR products. Proc Natl Acad Sci U S A 97:6640-5.
6. Pailler J, Aucher W, Pires M, Buddelmeijer N. 2012. Phosphatidylglycerol::prolipoprotein diacylglyceryl transferase (Lgt) of *Escherichia coli* has seven transmembrane segments, and its essential residues are embedded in the membrane. J Bacteriol 194:2142-51.
7. Legood S, Seng D, Boneca IG, Buddelmeijer N. 2022. A defect in lipoprotein modification by Lgt leads to abnormal morphology and cell death in *Escherichia coli* that is independent of major lipoprotein Lpp. J Bacteriol 204:e0016422.
8. Mychack A, Amrutha RN, Chung C, Cardenas Arevalo K, Reddy M, Janakiraman A. 2019. A synergistic role for two predicted inner membrane proteins of *Escherichia coli* in cell envelope integrity. Mol Microbiol 111:317-337.
9. Lehman KM, May KL, Marotta J, Grabowicz M. 2024. Genetic analysis reveals a robust and hierarchical recruitment of the LolA chaperone to the LolCDE lipoprotein transporter. mBio 15:e03039-23.
10. Bernhardt TG, de Boer PA. 2003. The *Escherichia coli* amidase AmiC is a periplasmic septal ring component exported via the twin-arginine transport pathway. Mol Microbiol 48:1171-82.
11. Mychack A, Janakiraman A. 2021. Defects in the first step of lipoprotein maturation underlie the synthetic lethality of *Escherichia coli* lacking the inner membrane proteins YciB and DcrB. J Bacteriol 203:e00640-20.
12. Guzman LM, Belin D, Carson MJ, Beckwith J. 1995. Tight regulation, modulation, and high-level expression by vectors containing the arabinose PBAD promoter. J Bacteriol 177:4121-30.
13. Weiss DS, Chen JC, Ghigo JM, Boyd D, Beckwith J. 1999. Localization of FtsI (PBP3) to the septal ring requires its membrane anchor, the Z ring, FtsA, FtsQ, and FtsL. J Bacteriol 181:508-20.
14. Lim HC, Bernhardt TG. 2019. A PopZ-linked apical recruitment assay for studying protein-protein interactions in the bacterial cell envelope. Mol Microbiol 112:1757-1768.
15. Mancia F, Love J. 2010. High-throughput expression and purification of membrane proteins. J Struct Biol 172:85-93.
16. Donnelly MI, Zhou M, Millard CS, Clancy S, Stols L, Eschenfeldt WH, Collart FR, Joachimiak A. 2006. An expression vector tailored for large-scale, high-throughput purification of recombinant proteins. Protein Expr Purif 47:446-54.
17. Amann E, Ochs B, Abel KJ. 1988. Tightly regulated tac promoter vectors useful for the expression of unfused and fused proteins in *Escherichia coli*. Gene 69:301-15.
